# Targeting CAMK1D-engineered nanoactivator suppresses cancer stem cell maintenance and immune evasion in enzalutamide-resistant prostate cancer

**DOI:** 10.7150/thno.120826

**Published:** 2026-01-01

**Authors:** Feifei Sun, Yuchuan Yan, Deqing Sun, Shijia Liu, Zhaoru Dong, Guoqiang Pan, Lin Zhang, Xianhao Shao, Yuliang Xu, Ying Qu, Tao Li

**Affiliations:** 1 Department of General Surgery, Qilu Hospital of Shandong University, Jinan, 250012, China.; 2 Department of Pharmacy, The Second Qilu Hospital of Shandong University, Jinan, 250033, China.; 3 Department of Pathology, Peking University People's Hospital, Beijing, China.; 4 Binzhou Center for Disease Control and Prevention, Binzhou, Shandong, China.; 5 Department of Orthopaedics, Shandong Provincial Hospital Affiliated to Shandong First Medical University, Jinan, Shandong, 250021, China.

**Keywords:** enzalutamide-resistant prostate cancer, CAMK1D, engineered lipid-based nanoactivator, stemness, immune evasion

## Abstract

**Rationale:** Hormonal therapy is fundamental to prostate cancer (PCa) management; however, its long-term efficacy is compromised by enzalutamide resistance (ENZR), which is fuelled by prostate cancer stem-like cells (PCaSCs) and an immunosuppressive microenvironment.

**Methods:** A CD44-targeted nanoactivator (EC@HNA) was engineered to co-deliver ENZ and siCAMK1D. Its physicochemical properties, cellular uptake and gene-silencing efficiency were characterized *in vitro*. Functional and mechanistic assays were used to assess PCaSCs expansion, cytokine modulation, immune cell dynamics, and CREB-dependent regulation of stemness genes. Therapeutic efficacy and safety were validated in ENZR cell cultures, murine tumor models, and patient-derived organoids.

**Results:** EC@HNA efficiently delivered siCAMK1D and achieved potent CAMK1D silencing, thereby significantly suppressing the expansion and self-renewal of PCaSCs. This treatment downregulated the immunosuppressive cytokines IL-10 and TGF-β, decreased regulatory T cell (Treg) infiltration, promoted M1-like polarization of tumor-associated macrophages, and enhanced CD8⁺ T cell infiltration and cytotoxicity in ENZR prostate tumors, thereby reprogramming the tumor immune microenvironment. Mechanistically, EC@HNA suppressed CREB phosphorylation at Ser133, which transcriptionally repressed key stemness regulators, including CD44, CD133, and NR4A1, thereby attenuating tumor stemness and immune evasion. These effects have been validated using *in vitro* cell models, ENZR xenografts, and patient-derived organoids. Collectively, EC@HNA dismantled the stemness-immunity axis sustaining ENZR and restored robust anti-tumor immunity with minimal systemic toxicity.

**Conclusions:** Overall, the CD44-targeted EC@HNA nanoplatform disrupted stemness programs and restored tumor-immune surveillance, representing a promising strategy to reverse ENZR and potentiate immunotherapy in clinical ENZR PCa patients.

## Introduction

Prostate cancer (PCa) remains a leading cause of cancer-related mortality in men, and enzalutamide (ENZ) serves as the cornerstone of anti-tumor treatment 1. Cellular heterogeneity is a well-recognized hallmark of advanced malignancies and provides a critical framework for understanding tumor aggressiveness and therapeutic resistance. Central to this framework is the cancer stem cell (CSCs) hypothesis, which proposes the existence of a subpopulation of tumor-initiating cells that are intrinsically resistant to conventional therapies 2, 3. CSCs possess the capacity for self-renewal and differentiation, thereby driving tumor heterogeneity and therapeutic resistance 4, 5. In PCa, CSCs can survive ENZ exposure and subsequently facilitate the repopulation of resistant tumor cells 5. Moreover, their ability to remain dormant during anti-tumor therapy poses a challenge for complete tumor eradication because these cells can later re-emerge and give rise to resistant tumors 6.

Mobilization of the immune system to recognize and eradicate tumor cells has evolved from a theoretical concept into a clinically effective therapeutic modality for various advanced cancers 7. Current strategies largely focus on suppressing pro-tumorigenic immune populations or boosting anti-tumor immunity, while the inherently immunosuppressive nature of tumor cells contributes to acquired resistance against immunotherapy; however, the molecular mechanisms underlying immune cell dysfunction within the tumor microenvironment remain incompletely defined 8. Notably, a high-stemness signature is associated with poor immunogenic responses, highlighting the potential correlation between stemness and immune evasion 9. CSCs not only mimic the characteristics of immune or vascular endothelial cells, promoting tumor growth and therapeutic resistance, but also facilitate immune escape by influencing immune and stromal cells through paracrine and juxtacrine pathways 7. Compared to the tumor bulk, CSCs possess the ability to reprogram their intrinsic signalling and rewire their interactions with immune cells in ways that impede therapeutic efficacy 10. Therefore, targeting PCaSCs represents a promising strategy to overcome immune evasion in enzalutamide resistant (ENZR) PCa and may enhance anti-tumor efficacy.

CAMK1D is a serine/threonine kinase that plays a critical role in regulating immune responses, cell proliferation, epithelial-mesenchymal transition (EMT), and survival 11-13. Notably, CAMK1D has emerged as a potential therapeutic target, especially in aggressive basal-like tumors, which share stem cell-like characteristics, hinting at its involvement in promoting stemness 14, 15. CAMK1D knockdown inhibits LNCaP-AI cell proliferation and migration, whereas its overexpression promotes tumor growth and confers androgen independence to LNCaP cells 16. Additionally, Volpin et al. demonstrated that CAMK1D is activated by cytotoxic T lymphocytes (CTLs) via Fas receptor stimulation, leading to the phosphorylation-dependent inhibition of caspase-3, -6, and -7 11. James et al. reported that CAMK1D overexpression impairs angiogenesis 17, whereas normalization of tumor vasculature improves tissue perfusion and immune infiltration, thereby augmenting the efficacy of immunotherapy 18.

**Figure 1:**
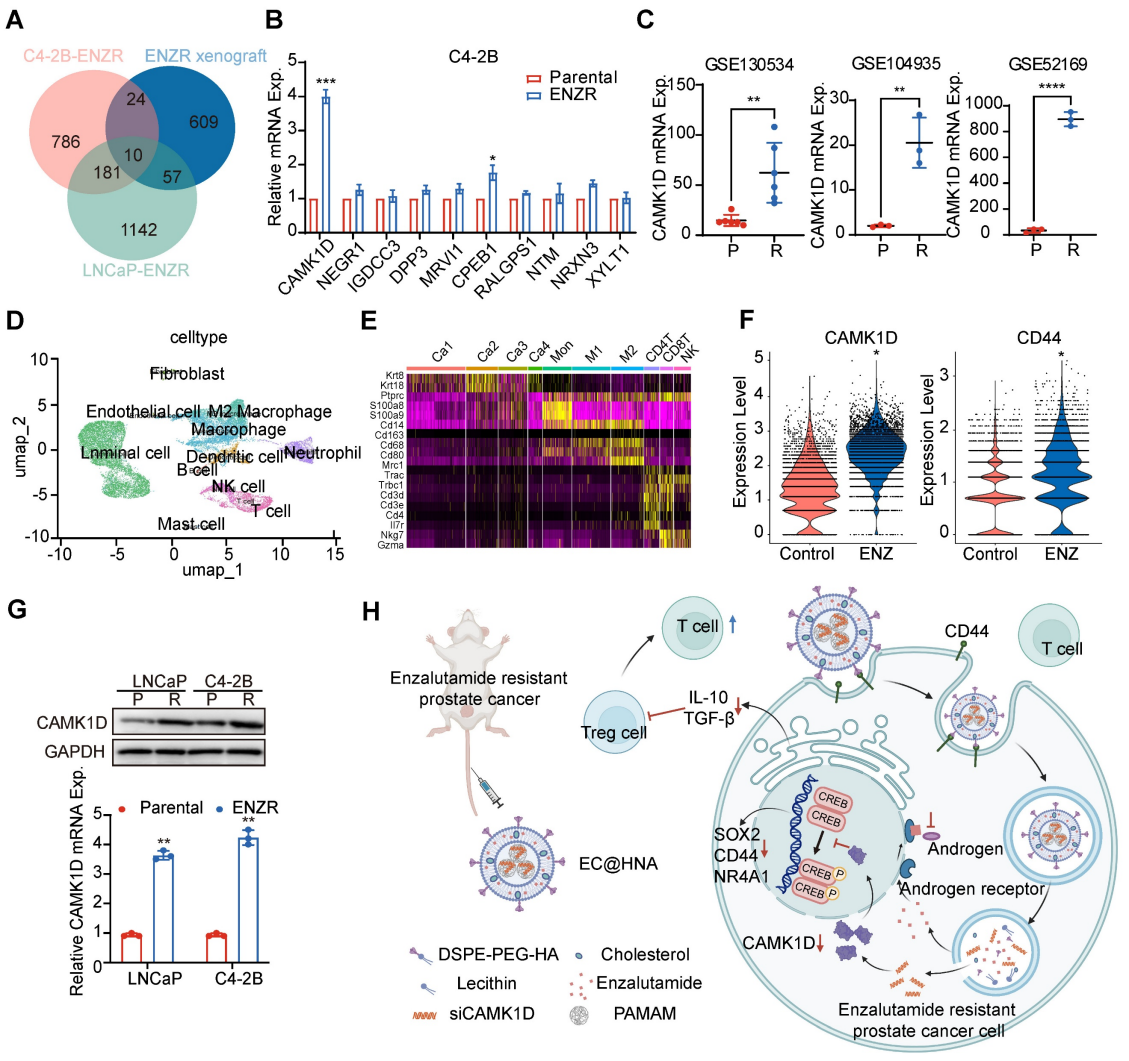
Identification and validation of CAMK1D as a key gene associated with ENZR. (A) Gene expression profiles associated with ENZR were systematically analyzed using publicly available transcriptomic datasets GSE110802, GSE55345, and GSE137833 from the GEO database. (B) Validation of ten candidate genes in previously established ENZ-sensitive and -resistant C4-2B cells. mRNA expression levels were quantified using RT-qPCR. (C) CAMK1D expression analysis in public ENZR datasets. P, parental; R, ENZR. (D, E) Integrative scRNA-seq analysis of mouse PCa samples (GSE299843), visualized using a unified UMAP embedding for cell annotation. Ca1-4, cancer cell clusters 1-4. (F) scRNA-seq analysis of CAMK1D and CD44 expression in ENZ-treated versus control groups. (G) CAMK1D expression levels in ENZ-sensitive and -resistant LNCaP and C4-2B cells, assessed via RT-qPCR and western blot. Resistant cells were maintained in medium containing 10 μM ENZ. P, parental; R, ENZR. (H) Schematic illustration of EC@HNA-mediated targeting of CAMK1D in ENZR PCa. Data distribution was assessed using the Shapiro-Wilk test, and statistical significance was determined using a two-sided Student's *t*-test. ^*^P < 0.05, ^**^P < 0.01, ^****^P < 0.0001.

Therefore, we engineered a hyaluronic acid-modified lipid-based nanoactivator (EC@HNA) for the co-delivery of ENZ and siCAMK1D to enhance the sensitivity of ENZR cells to T cell-mediated cytotoxicity, while mitigating immune evasion driven by stemness-related factors. EC@HNA effectively silenced CAMK1D expression and inhibited the self-renewal capacity of ENZR cells by reducing CREB phosphorylation at Ser133. Moreover, EC@HNA reprogrammed the immune microenvironment by lowering IL-10 and TGF-β levels, thereby decreasing Treg cell accumulation, increasing CD8+ T cell infiltration, and ultimately retarding the progression of ENZR PCa (Figure 1H).

## Materials and Methods

### PCa samples

We selected cases with ENZ treatment durations exceeding 3 months. One case presented with an initial PSA level of 16.3 ng/mL, while another had a PSA level of 0.28 ng/mL at the start of treatment. For organoid culture, we prioritized cases demonstrating minimal histological response, defined as a reduction in tumor cells between 10% and 30%. The study was approved by the Institutional Review Board of Shandong University (Document No. KYll-2022(ZM)-1359, date: January 1, 2024) and conducted following international ethical guidelines.

### Cell lines

Human PCa cell lines LNCaP (CVCL_0395), C4-2B (CVCL_4784), 22Rv1(CVCL_1045), as well as the human embryonic kidney cell line HEK293T (CVCL_0063), and murine PCa cell lines Myc-CaP(CVCL_J703), RM-1 (CVCL_B459), were obtained from the the American Type Culture Collection (ATCC) and cultured according to the manufacturer's instructions. The LNCaP-ENZR and C4-2B-ENZR cell lines were established as previously described 19 and were cultured in the presence of 10 µM ENZ (Energy Chemical; catalog no. E120461). For *in vivo* studies, the intrinsically ENZR murine PCa cell line RM-1 was utilized to investigate mechanisms of resistance and therapeutic responses, as previously reported 20. All cell lines were routinely tested and confirmed to be free of mycoplasma contamination (Beyotime; catalog no. C0298S). Additionally, the cumulative culture duration from thawing to experimental use did not exceed 15 passages.

### Construction of siCAMK1D-NP (cNP)

The siCAMK1D (Human, SR311360; mouse, SR412965) was complexed with PAMAM (Sigma, USA) at varying weight ratios (1:1, 1:2, 1:4, 1:8, 1:10, and 1:20) in pH 7.4 phosphate buffer. The resulting cNP complexes were analyzed by electrophoresis on a 2% (w/v) agarose gel for 40 min at 60 V and room temperature, followed by UV imaging using a UV illuminator (Tanon, China). The knockdown efficiency of PAMAM-delivered siCAMK1D was evaluated by immunofluorescence and western blot analysis.

### Construction of EC@HNA

Phospholipid (Aladdin, China), cholesterol (J&K, China), DSPE-PEG-HA (Chongqing Yusi Pharmaceutical Technology Co., Ltd., China), and ENZ (Energy, China) were dissolved in ethanol and subsequently introduced to ultrapure water, stirring at 30 ℃ for 4 h to promote ethanol evaporation. cNP complexes were then added and stirred at room temperature for an additional 30 min, yielding the lipid-based nanoparticles, EC@HNA, co-loaded with ENZ and siCAMK1D.

### Gel retardation assay

To evaluate the protective effect of HNA on siCAMK1D, samples were treated with RNase or PBS at 37 ℃ for 1 h. The samples were then analyzed by gel electrophoresis, and the resulting gels were visualized under a UV illuminator to determine the localization of siCAMK1D.

### Cellular uptake* in vitro*

C4-2B-ENZR and RM-1 cells were incubated overnight with siCAMK1D+FITC, FC@NA or FC@HNA (Cy3-labeled siCAMK1D 20 nM, FITC 200 ng/mL). Cellular uptake was then assessed using a confocal laser scanning microscope (CLSM) and quantified by flow cytometry.

### Endolysosomal escape

C4-2B-ENZR and RM-1 cells were incubated with C@HNA at 37 °C for 1, 3, and 6 h (Cy3-labeled siCAMK1D 20 nM). After treatment, cells were incubated with LysoTracker Green at 37 ℃ for 30 min and subsequently fixed with 4% paraformaldehyde. Cells were stained with DAPI and analyzed using CLSM. For quantitative assessment of co-localization between C@HNA and LysoTracker Green, Manders' co-localization coefficients were calculated using ImageJ.

### Hemolysis test

Whole blood was collected from rabbits, and erythrocytes were isolated through centrifugation. After being washed twice with PBS, the erythrocytes were co-cultured with HNA, EC@HNA, normal saline, or H₂O for 3 hours. The supernatants were then collected, and UV-visible absorbance at 570 nm was measured using a microplate reader to evaluate the hemolysis rate.

### *In vitro* drug release studies

The *in vitro* release of ENZ from ENZ solution or EC@HNA was evaluated using dialysis membranes (MWCO 3500 Da). ENZ solution or E@HNA (equivalent to 950 µg of ENZ) were sealed in dialysis bags and immersed in 40 mL of PBS (pH 7.4, containing 5% Tween 80) at 37 °C with gentle shaking at 150 rpm. At predetermined intervals, 0.5 mL of the medium was withdrawn and replaced with an equal volume of fresh buffer. ENZ concentrations were quantified by HPLC (Agilent 1260 Infinity II, C18 Luna column, 100 × 4.6 mm) using an ammonium acetate buffer/acetonitrile (40:60, v/v) mobile phase at 0.8 mL min⁻¹, with detection at 270 nm.

For siCAMK1D release analysis, free Cy5.5-labeled siCAMK1D or C@HNA (equivalent to 11 µg of Cy5.5-labeled siCAMK1D) was dispersed in 10 mL PBS (pH 7.4) and incubated at 37 °C under shaking. At scheduled time points, 1 mL of the medium was collected and replenished with fresh buffer. The collected samples were centrifuged, and the fluorescence intensity of the supernatant was measured using a microplate reader to determine the cumulative release of siCAMK1D.

### *In vivo* biodistribution of DC@HNA

PCa subcutaneous tumor models were intravenously injected with DiR+siCAMK1D(Cy3), DiR-siCAMK1D(Cy3)@NA (DC@NA), DC@HNA (containing 15 µg of Cy3-labeled siCAMK1D and 10 µg of DiR per mouse), and the fluorescence signals of DiR and Cy3 were monitored throughout the body using an In Vivo Imaging System (IVIS) imaging system at designated time points (0, 1, 4, 6, 12, 24, and 48 h). After 48 h, the mice were euthanized, and biodistribution of DC@HNA in various organs was evaluated via IVIS imaging. Data analysis was performed using Living Image software.

### Synergy analysis

The synergistic effects of siCAMK1D and ENZ were evaluated using the SynergyFinder 2.0 platform (https://synergyfinder.org/). LNCaP-ENZR and C4-2B-ENZR cells were exposed to serial dilutions of ENZ, siCAMK1D, or their combinations for 72 h, followed by CCK8 assay. The ZIP model was applied to calculate synergy scores, where a score > 10 indicates synergism.

### RNA extraction and quantitative real-time PCR (RT-qPCR)

RT-qPCR was utilized to assess CAMK1D mRNA expression in C4-2B-ENZR, LNCaP-ENZR, 22Rv1 and RM-1 cells following treatment with various formulations. Total RNA was extracted using Trizol reagents (Invitrogen; catalog no. 15596026) and reverse-transcribed into cDNA using the First Strand cDNA Synthesis Kit (Toyobo; catalog no. FSQ-201) according to the manufacturer's instructions. mRNA levels were quantified by a SYBR Green PCR kit (Roche; catalog no. 04887352001), with primer sequences detailed in Table S2. Relative mRNA levels were calculated using the 2^-ΔCt^ method, with GAPDH serving as an endogenous control.

### Western blot and immunohistochemistry (IHC)

Western blot and IHC were performed as previously described procedures 21. Details of the primary antibodies used in this study are provided in Table S1. The IHC slides were independently and blindly evaluated by two experienced pathologists (Q.M. and B.H.) to assess the expression of CAMK1D, CD44, CD133 and Ki67.

### Cellular viability, proliferation and clone formation assays

Cells were seeded in 96-well plates at a density of 2,000 cells/well for LNCaP-ENZR and C4-2B-ENZR lines, and 500 cells/well for RM-1 and Myc-CaP cells for overnight attachment. The culture medium was replaced with 100 μL fresh medium containing 10 μL CCK-8 reagent. Cells were incubated for 2 h at 37 ℃, and the absorbance at 450 nm was determined using a Multiskan FC microplate reader (Thermo Fisher Scientific). Colony formation assays were employed to evaluate cell clonogenic capacity. For clone formation assays, treated cells were seeded in 6-well plates, subjected to the ENZ treatment (10 µM), and then fixed, stained, and counted after approximately 15 days.

### Matrigel 3D culture

Dissociated cells were cultured in RPMI-1640 medium supplemented with B-27 (1:50; Gibco, catalog no. A3582801), bFGF (20 ng/mL; Corning, catalog no. 354060), and EGF (40 ng/mL; Corning, catalog no. 354052). For Matrigel culture, 50 μL of Matrigel was added to each well of a 6-well plate and solidified at 37 °C for 30 min. The cell suspension (100 μL) was then mixed with an equal volume of cold Matrigel and overlaid onto the preformed Matrigel layer. After incubation at 37 °C for 30 min, 2.5 mL of prewarmed RPMI-1640 medium was added to each well. Cells were maintained for 10-14 days with a 50% medium replacement every three days.

### Co-Immunoprecipitation (Co-IP)

Co-IP assays were performed according to the manufacturer's instructions provided with the Pierce™ Co-IP Kit (Thermo; catalog no. 26149). The resulting immunocomplexes were collected and analyzed by western blot. For the western blot assays, primary antibodies used included Flag (1:100, Cell Signaling Technology; catalog no. 14793S) and CREB (1:100, Proteintech; catalog no. 12208-1-AP).

### Fluorescence resonance energy transfer (FRET)

To evaluate protein-protein interactions, FRET assays were performed. Human HEK 293T cells were transfected with plasmids encoding cyan fluorescent protein (CFP)-CAMK1D and yellow fluorescent protein (YFP)-CREB. After a 24 h incubation on glass coverslips, the cells were fixed with 4% paraformaldehyde, followed by immunocytochemical analysis to assess protein interactions. FRET efficiency was quantified based on histograms generated from the analysis of over 15 cells per experimental condition, and each experimental condition was performed in triplicate to ensure consistency and reproducibility.

### Molecular docking

The crystal structures of CAMK1D (UniProt ID: Q8IU85) and CREB (UniProt ID: P16220) were retrieved from the AlphaFold database (https://alphafold.com). The central coordinates for molecular docking were determined using GRAMM Docking (https://gramm.compbio.ku.edu). Virtual molecular docking was subsequently performed using AutoDock Vina. The optimal binding conformation was visualized with PyMOL (version 2.5.7), and the docking images were generated for further analysis.

### Immunofluorescence staining

For immunofluorescence analysis, 4 μm tissue sections were fixed and incubated to staining with primary antibodies against CAMK1D (1:100, Abcam, catalog no. ab198165), CD44 (1:100, Proteintech, catalog no. 15675-1-AP), CD133 (1:100, Proteintech, catalog no. 18470-1-AP), CREB (1:100, Proteintech, catalog no. 12208-1-AP; 1:100, Abcam, catalog no. ab178322), and P63 (1:600, Abcam, catalog no. ab124762). After primary antibody incubation, tissue sections were processed using a four-color multiplex immunofluorescence staining kit (HUILAN Bio, catalog no. RC0086-34RM), whereas cells were incubated with fluorescently labeled secondary antibodies. Nuclei were counterstained with DAPI, and images were captured using a confocal microscope for detailed visualization.

### Single cell RNA-sequencing (scRNA-seq) analysis

We obtained PCa tissue samples from a pair of mice for scRNA-seq. During cell sorting, immune cells were enriched using the cell Isolation Kit II (Miltenyi Biotec; catalog no. 130-095-130) and subsequently stained with CD45 antibody (BD Pharmingen; catalog no. 553079). The sequencing data have been deposited in the Gene Expression Omnibus (GEO) under accession number GSE299843. To enable cross-species comparison and validation in human datasets, we also retrieved publicly available scRNA-seq data from the GEO database (https://www.ncbi.nlm.nih.gov/geo/), including GSE137829 22. After quality control of the raw data, normalization was performed to ensure comparability across cells, followed by dimensionality reduction using UMAP to visualize cellular heterogeneity. Cell type clustering was subsequently conducted to define distinct cellular populations. Differentially expressed genes (DEGs) between PCa tumor cells and associated immune cells were identified, and Gene Ontology (GO) and Kyoto Encyclopedia of Genes and Genomes (KEGG) enrichment analyses were performed to elucidate the biological pathways and functional processes involved.

### Patient-derived organoids

Human PCa samples were collected and preserved in tissue storage solution (Miltenyibiotec; catalog no. 130-100-008) at 4 °C. The tissues were washed three times with RPMI-1640 medium containing penicillin and streptomycin, and necrotic areas were carefully removed. The remaining tissue was finely minced using sterile ophthalmic scissors. The bulk-isolated prostatic cells were then embedded in 50 µl droplets of basement membrane extract (Corning; catalog no. 356255) and subsequently cultured in human prostate organoid medium (MasterAim, catalog no. 10-100-029).

### RNA sequencing

For RNA sequencing, CAMK1D gene knockdown was achieved in C4-2B-ENZR cells. Following confirmation of the knockdown efficiency and completion of relevant functional assays, 1 × 10^6 cells per sample were lysed in Trizol and stored on dry ice for subsequent sequencing analysis.

### Animal models

All animal experiments were conducted in accordance with the welfare and ethical guidelines established by the Experimental Animal Welfare and Ethics Committee of Qilu Hospital of Shandong University (Document No. KYll-2022(ZM)-1359, date: January 1, 2024). Mice were housed and maintained at the Animal Management Center of Shandong University under standard conditions. RM-1 cells were orthotopically injected into the prostate tissue of C57BL/6 mice at a concentration of 1 × 10^5 cells per mouse. *In vivo* imaging on day 7 confirmed tumor formation, with fluorescence intensity reaching ~10 counts. At this stage, mice were randomly assigned to treatment groups and received various formulations of siCAMK1D (3 μg for each mouse) or/and ENZ (5 mg/kg) via tail vein injection every three days. Tumor fluorescence intensity was measured on days 0, 4, 7, 12, 14, and 21. On day 21, mice were sacrificed, and tumors tissues were collected for further analyses. To evaluate the effects of treatment on survival, orthotopic PCa models were established using the same procedure, followed by the indicated treatments, and survival was monitored for up to 60 days to generate Kaplan-Meier survival curves. Throughout the study, tumors growth was carefully monitored to ensure that the maximum tumor diameter did not exceed 15 mm.

### Flow cytometry analysis

Surface staining of mouse cells was performed using the following fluorescent-conjugated antibodies: CD45 (BD Pharmingen; catalog no. 553079), CD3 (BD; catalog no. 557596), CD8 (BD Pharmingen; catalog no. 551162), F4/80 (Biolegend; catalog no. 123137), CD86 (Biolegend; catalog no. 159203) and CD206 (Biolegend; catalog no. 141707). All samples were acquired on a BD Fortessa and analyzed with FlowJo software.

### Apoptosis

ENZR PCa cells were treated with Control, HNA, C@HNA, E@HNA, or EC@HNA (containing 5 µM ENZ or/and 50 nM siCAMK1D) for 48 h. Apoptosis was assessed using an Annexin V-FITC/propidium iodide (PI) detection kit (C1062; Beyotime, China) according to the manufacturer's instructions. The proportion of apoptotic cells was analyzed with a Beckman Coulter FC500 flow cytometer (Brea, CA, USA), and data were processed using FlowJo software.

### Chromatin immunoprecipitation (ChIP)

Chromatin from pretreated cells was fixed with 1% formaldehyde for 11 min. DNA was sheared to fragments approximately 200-500 bp using a sonicator (10^6 cells in 500 μL volume; ultrasound for 15 s, pause for 9 s, repeated five times). Chromatin immunoprecipitation was performed using anti-CREB antibodies (Abcam, Cambridge, MA, USA) and anti-IgG antibodies (St. Louis, MO, USA). The enrichment of CREB within SOX2, CD44, BCL2 and NR4A1 promoter was assessed by RT-PCR using chromatin immunoprecipitated from cells with the indicated primers listed in Table S2.

### Statistical analysis

The experiments were independently repeated at least three times with consistent results. Data are presented as mean ± SD. Normality was assessed visually using histograms and boxplots. Categorical variables are presented as frequency and percentage. For normally distributed data, Student's t-test was used; otherwise, the Mann-Whitney U test was applied. Survival analysis was performed using the Kaplan-Meier method with Log-rank tests. Statistical analyses were conducted with GraphPad Prism 9 software (RRID: SCR_002798), and correlations were assessed using the Chi-Square Test or Fisher's exact test. Tumor growth data were analyzed by ANOVA, with significance set at *P* < 0.05.

## Results

### CAMK1D mediates ENZR *in vitro* and *in vivo*

To elucidate the molecular mechanisms underlying ENZR in PCa, we performed a comprehensive analysis of resistance-associated databases, including C4-2B-ENZR, C4-2B-ENZR xenografts, and LNCaP-ENZR and identified several significantly upregulated genes associated with ENZR (Figure 1A). RM-1 cells were used as a natural ENZR PCa model. RM-1 cells exhibited intrinsic resistance to ENZ (IC₅₀: 51.7 μM), approximately seven-fold higher than that of androgen-dependent Myc-CaP cells (IC₅₀: 7.19 μM), indicating a stable ENZR phenotype suitable for investigating intrinsic resistance mechanisms in PCa. The expression of these genes was further validated in ENZR cell lines (Figures 1B, and S1A-B). Among them, CAMK1D was markedly upregulated in ENZR cells compared to that in control cells, a finding corroborated by analysis of public databases (Figure 1C). Single-cell transcriptomic analysis demonstrated a significant increase in CAMK1D and CD44 expression in the ENZ-treated group compared to that in controls (Figure 1D-F). Moreover, analysis of single-cell transcriptomic data from human neuroendocrine prostate cancer (NEPC) revealed a marked elevation in CAMK1D expression within the stemness-associated cluster (Figure S2). Consistently, CAMK1D expression was significantly elevated in ENZR cells compared to their parental counterparts (Figure 1G). Moreover, prolonged ENZ treatment (approximately six months) led to a sustained increase in CAMK1D expression by PCa cells (Figure S3A-B). Collectively, these findings established CAMK1D as a consistently upregulated gene in ENZR, highlighting its potential role in resistance mechanisms (Figure 1H).

### Preparation and characterization of EC@HNA

**Figure 2:**
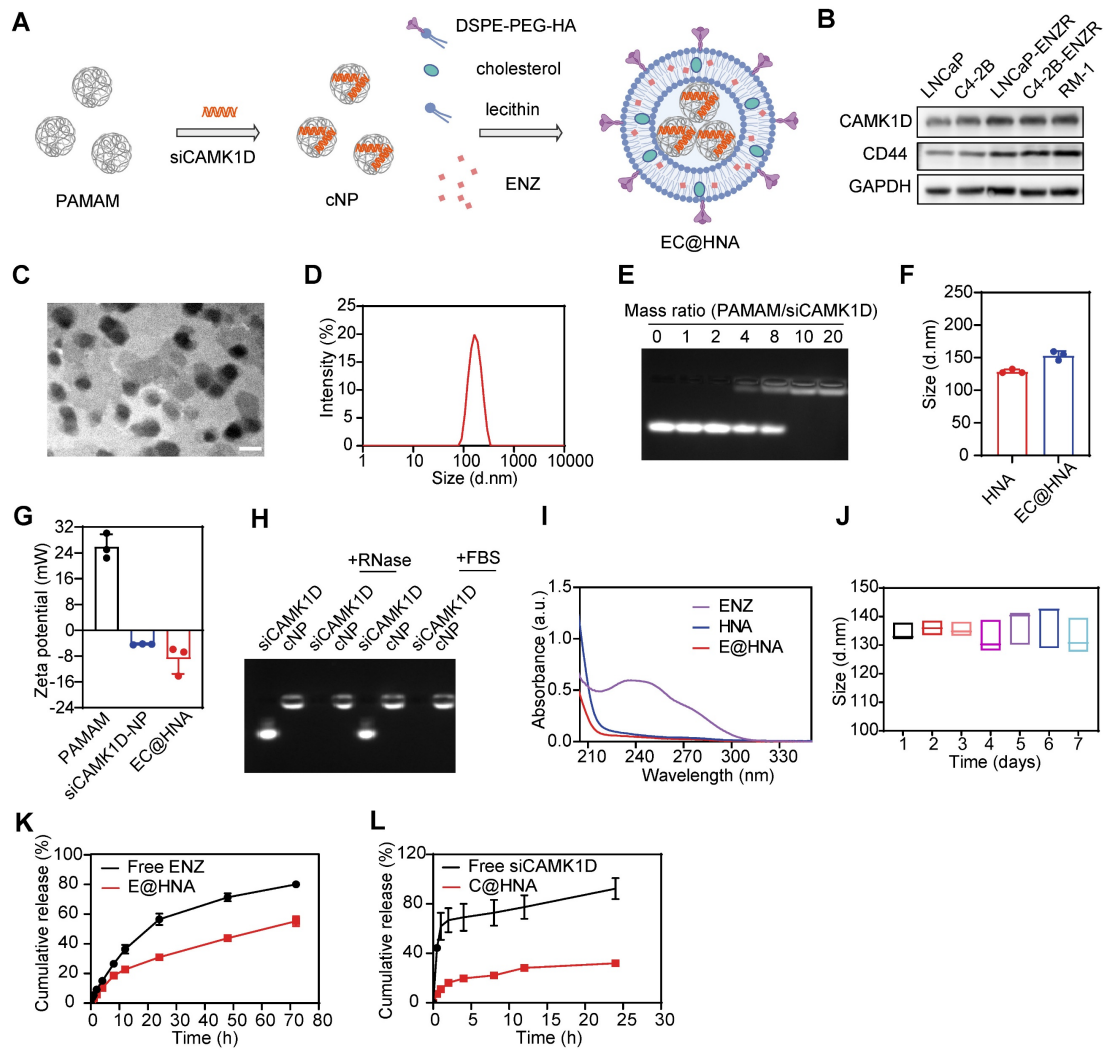
Preparation and characterization of EC@HNA. (A) Schematic illustration of the preparation process for ENZ and siCAMK1D co-entrapped nanoactivator (EC@HNA). (B) Western blot analysis of CAMK1D and the stemness marker CD44 in PCa cells. (C) Transmission electron microscopy image of EC@HNA showing a well-defined spheroid structure. Scale bar, 150 nm. (D) Hydrodynamic size distribution of EC@HNA determined by dynamic light scattering (DLS). (E) Agarose gel electrophoresis demonstrating siRNA binding stability in cNP complexes at different mass ratios. (F) DLS measurement of particle size variations upon EC@HNA assembly. (G) Zeta potential changes of PAMAM, cNP, and EC@HNA assessed by DLS. (H) Gel retardation assay confirming siCAMK1D stability in EC@HNA after RNase and fetal bovine serum (FBS) treatment at 37 ^o^C for 1 h. (I) UV absorption spectra of ENZ, HNA, and EC@HNA, validating successful drug loading. (J) Stability assessment of EC@HNA in PBS at 4 ^o^C over 7 days. (K) ENZ and (L) siCAMK1D release profiles of EC@HNA in PBS (pH 7.4) over time. Data are presented as mean ± SD.

Small interfering RNA (siRNA)-based gene therapies hold great promise for drug development. However, naked siRNA is rapidly degraded by RNases and cannot efficiently cross cell membranes because of its negative charge, leading to poor transfection efficiency and limited therapeutic efficacy 23. To enhance targeted delivery, we employed a G5-PAMAM dendrimer as a gene vector for siRNA loading and incorporated it into lipid nanoparticles for effective siRNA transport (Figure 2A). The cancer stemness markers CD44 and CD133 are key mediators of drug resistance, driving tumor survival and poor treatment responses 4. CD44, a broadly expressed CSC marker, serves as a central regulator of cell adhesion, migration, stemness maintenance, and immune modulation, and represents a promising therapeutic target in tumor progression and treatment resistance 24. We confirmed that CD44 expression was significantly upregulated in ENZR PCa cells (Figure 2B). Given the strong binding affinity of hyaluronic acid (HA) for CD44, HA was selected as a targeting ligand to enhance the specificity of the co-delivery system for ENZR PCa cells.

Transmission electron microscopy revealed that EC@HNA exhibited a well-defined spheroid morphology with an average particle size of 125.2 ± 2.3 nm (Figure 2C-D). EC@HNA complexes were prepared at various mass ratios via self-assembly, with agarose gel electrophoresis confirmed the stable binding of siRNA to G5-PAMAM at ratios > 10:1 (Figure 2E). Fluorescence imaging and western blot analyses demonstrated that optimal transfection efficiency was achieved at a PAMAM/siCAMK1D mass ratio of 10:1 (Figure S4A-B), consistent with the gel retardation results. Moreover, CCK-8 assays confirmed that the applied concentrations of G5-PAMAM and HNA caused no significant cytotoxicity (Figure S4C). Upon the cNP and ENZ loading, the particle size increased, accompanied by corresponding changes in the zeta potential and UV absorption spectra, verifying the successful construction of EC@HNA (Figure 2F-H). Based on the mass ratio, the N/P ratio of PAMAM to siCAMK1D was 14:1. The drug-loading capacity and encapsulation efficiency of ENZ by HNAs were 2.1% and 99.1%, respectively. For siCAMK1D, the loading performance is typically expressed as the N/P ratio between PAMAM and siCAMK1D, which was 14:1, corresponding to a drug-loading capacity of 0.49% and an encapsulation efficiency of 95.3% for HNA. These results confirmed that HNA possesses excellent drug-carrying capability and meets the criteria for an effective nanocarrier system.

G5-PAMAM provided effective protection against RNase degradation, as siCAMK1D complexed within EC@HNA remained stable in the presence of 0.1 mg/mL RNase for 1 h, whereas naked siCAMK1D was rapidly degraded (Figure 2I). Furthermore, CCK-8 and hemolysis assays demonstrated the biocompatibility of HNA, showing no significant impact on cell viability and < 5% hemolysis in rat red blood cells at therapeutic doses (Figure S4D-F). Stability tests further confirmed that EC@HNA remained structurally intact in PBS at 4 °C for at least 7 days, underscoring its suitability for the co-delivery of gene therapies and small-molecule drugs (Figure 2J). Under physiological conditions, the cumulative 24 h release of free ENZ and siCAMK1D reached 57.11% and 92.37%, respectively. In contrast, EC@HNA exhibited markedly slower release kinetics, with 31.95% of ENZ and 30.99% of siCAMK1D released within 24 h, indicating excellent formulation stability under physiological conditions (Figure 2K-L). These results demonstrated that EC@HNA effectively prolonged the release of both agents, which is critical for maintaining drug stability and achieving sustained therapeutic effects.

**Figure 3:**
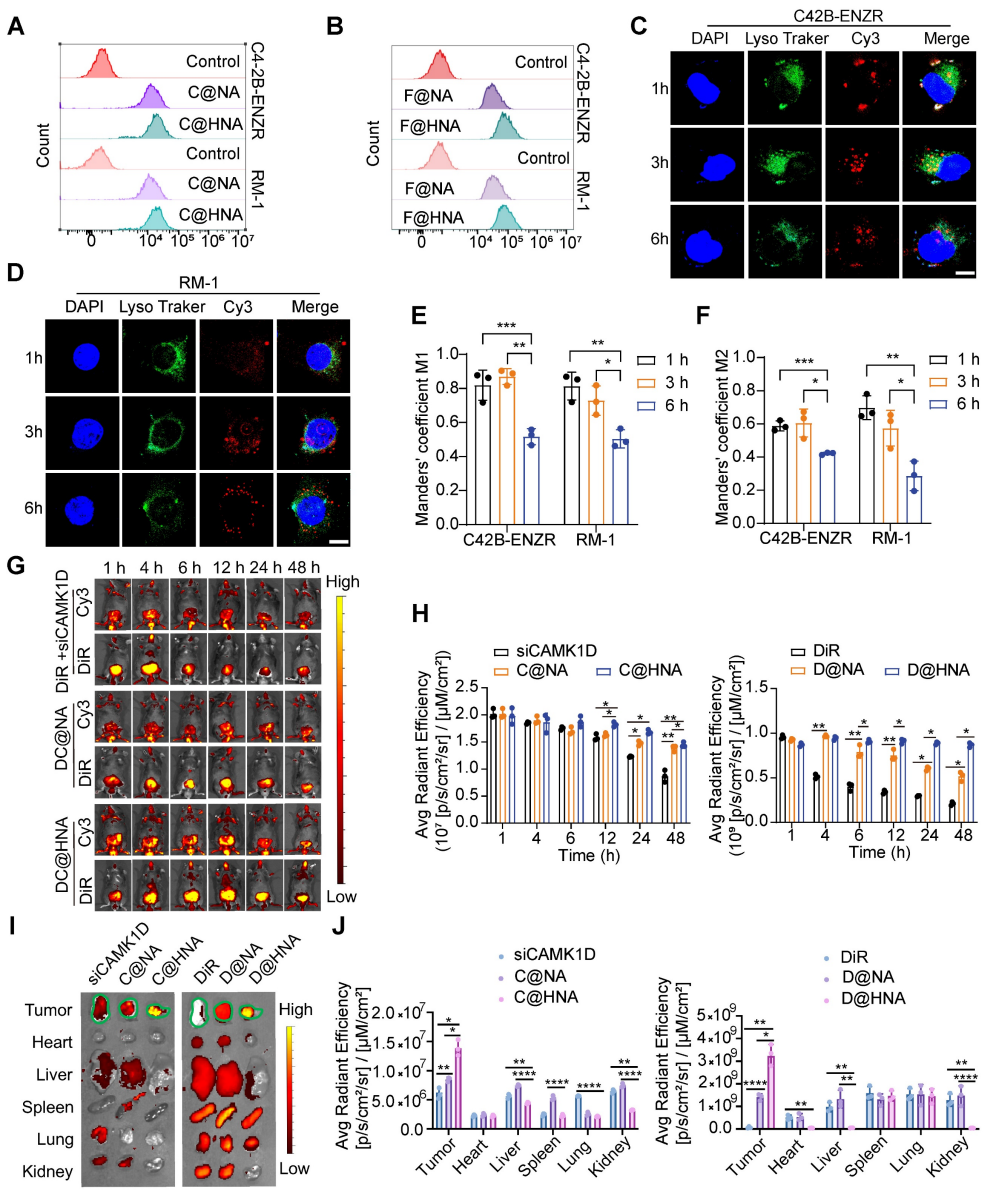
Enhanced drug delivery, cellular uptake, and efficient lysosomal escape of HNA. (A, B) Flow cytometric analysis of cellular uptake of HNA or NA by ENZR PCa cells, as measured by confocal microscopy with siCAMK1D labeled with Cy3 and ENZ replaced with FITC. (C, D) Confocal images illustrating the subcellular localization of C@HNA and lysosomes in C4-2B-ENZR and RM-1 cells after incubation at 37 ^o^C for 1, 3, and 6 h. Scale bar, 10 µm. (E, F) Quantitative analysis of co-localization of Cy3-labeled siCAMK1D and LysoTracker Green-labeled endo/lysosomes (n=3). (G, H) *In vivo* fluorescence imaging using IVIS and quantitative analysis following tail vein injection of siCAMK1D+DiR, DC@NA, and DC@HNA in mice (n=3). (I) Images and (J) quantification of fluorescence in major organs (heart, liver, spleen, lung, and kidney) and tumors 48 h after administration of siCAMK1D+DiR, DC@NA, and DC@HNA (n=3). Data are presented as mean ± SD. ^*^P < 0.05,^ **^P < 0.01, ^***^P < 0.001, ^****^P < 0.0001.

We further verified the transfection efficiency and targeting specificity of EC@HNA. After 4 h of incubation, the Cy3 and FITC signals were significantly higher in C4-2B-ENZR and RM-1 cells treated with FC@HNA than in those treated with FC@NA, indicating enhanced cellular uptake mediated by HA targeting (Figures 3A-B, and S5A-B). In C4-2B-ENZR and RM-1 cells treated with FC@HNA, the Cy3 mean fluorescence intensity (MFI) increased by 1.23-fold and 1.14-fold, respectively, compared to that in the FC@NA group, and the corresponding FITC fluorescence signals were elevated by approximately 2.44-fold and 2.26-fold (Figure S5C-D).

Furthermore, in C4-2B-ENZR cells treated with C@HNA, the overlap between Cy3 and LysoTracker Green progressively decreased over time, suggesting efficient endo/lysosomal escape of C@HNA (Figure 3C-F). To evaluate *in vivo* biodistribution, siCAMK1D+DiR, DC@NA, and DC@HNA were administered via tail vein injection. The results showed that the Cy3 signal at the orthotopic prostate tumor site was markedly higher in the C@HNA group than in the blank control group, which may be attributed to the orthotopic tumor shifting closer to the ventral skin (Figure S5E-F). The DC@HNA group exhibited significantly higher Cy3 and DiR fluorescence intensities than the siCAMK1D+DiR and DC@NA groups, demonstrating efficient tumor accumulation of the nanoactivator. Notably, at 48 h post-injection, the DC@HNA group retained the strongest fluorescence signals, highlighting enhanced tumor targeting and prolonged retention (Figure 3G-H). *In vivo* imaging further confirmed the highest fluorescence intensity at tumor sites in DC@HNA-treated mice compared to those receiving siCAMK1D+DiR or DC@NA (Figure 3I-J). Overall, these findings demonstrate that EC@HNA effectively targets ENZR PCa, enhances the intracellular delivery of chemical drugs and gene therapies, and facilitates cytosolic siRNA release, underscoring its potential for advanced biomedical applications.

### CAMK1D promotes stem-like properties in PCa cells

**Figure 4:**
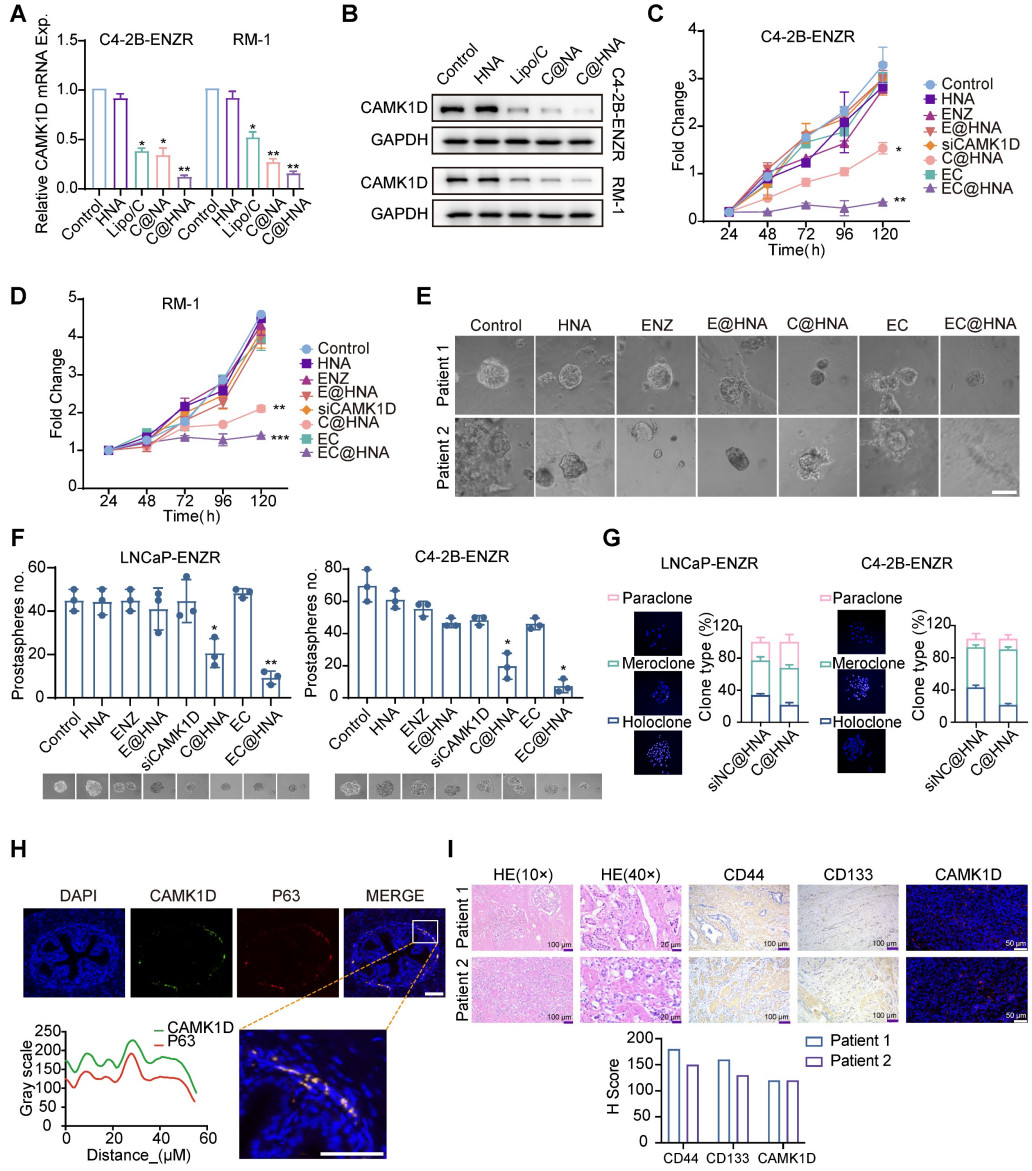
Correlation between CAMK1D expression and stemness phenotype in PCa. (A, B) C4-2B-ENZR and RM-1 cells were transfected with HNA, Lipo3000/siCAMK1D (Lipo/C), C@HNA, or EC@HNA (siCAMK1D: 50 nM; ENZ: 5 μM). CAMK1D mRNA and protein expression levels were assessed using RT-qPCR and western blot, respectively. (C, D) Cell proliferation of LNCaP-ENZR and C4-2B-ENZR was evaluated by CCK-8 assays after treatment with HNA, ENZ, E@HNA, siCAMK1D, C@HNA, EC, or EC@HNA (siCAMK1D: 50 nM; ENZ: 5 μM). (E) Representative microscopic bright-field images of different human PCa organoids treated with PBS (control), HNA, ENZ, E@HNA, C@HNA, EC, or EC@HNA (siCAMK1D: 50 nM; ENZ: 5 μM). Scale bar, 50 μm. Cases with treatment durations > 3 months were selected, with PSA levels of 16.3 ng/mL and 0.28 ng/mL at the time of treatment in two representative cases. (F) ENZR cells were cultured in low-attachment plates to form 3D spheroids and treated with the indicated reagents. (G) Clonogenic assays were performed in ENZR cells following CAMK1D knockdown. Representative images of three types of clones (left) and quantification (right) are shown. (H) Immunofluorescence staining and quantification showing CAMK1D (green) and P63 (red) localization in mouse prostate tissue. (I) HE staining and protein expression of CD44, CD133, and CAMK1D in two cases used for organoid culture. Data were assessed for normal distribution using the Shapiro-Wilk test. The two-sided Student's *t*-test was used for comparison among variables. ^*^P < 0.05, ^**^P < 0.01, ^***^P < 0.001.

To investigate the impact of CAMK1D targeting on ENZR cell stemness, we first assessed the delivery efficiency of HNA into PCa cell lines and confirmed that EC@HNA achieved the highest CAMK1D knockdown efficiency, outperforming conventional transfection reagents (Figures 4A-B and S6A-B). CCK-8 assays further demonstrated that EC@HNA-mediated CAMK1D depletion significantly suppressed the proliferation of ENZR cells, whereas NP alone had no discernible effect (Figures 4C-D and S6B-D). To further elucidate the biological effects of EC@HNA, we established organoid cultures from patients exhibiting poor responses to ENZ and demonstrated that EC@HNA treatment markedly impaired clonogenic growth compared with the other groups (Figure 4E). 3D spheroid formation assays demonstrated that ENZR cells treated with EC@HNA exhibited markedly reduced growth compared with the other groups (Figure 4F). Clonal tracing studies have indicated that holoclones contain long-lived stem cells with extensive self-renewal and progenitor generating capacities 25. Consistently, we observed high coexpression of CAMK1D and CD133 in the holoclones, which was significantly diminished by CAMK1D inhibition (Figure 4G). Notably, cells located in the proximal region of the mouse prostate, particularly within the basal compartment, display stem-like properties characterized by self-renewal, multilineage differentiation, and tissue regeneration 26. Consistently, these proximal cells exhibited elevated CAMK1D expression and co-expression of the basal cell marker P63 (Figure 4H). Furthermore, the expression of CAMK1D in organoid models colocalized with the stemness markers CD44 and CD133 (Figure 4I). To assess the synergistic effects of siCAMK1D and ENZ, CCK-8 assays were performed. SynergyFinder analysis yielded ZIP synergy scores of 11.83 in LNCaP-ENZR cells and 16.4 in C4-2B-ENZR cells, indicating a clear synergistic interaction between the two agents. (Figure S6F-I). Collectively, these results establish that CAMK1D is a key regulator of ENZR cell stemness.

### Evaluation of EC@HNA for overcoming ENZR in *in situ* tumor models

**Figure 5:**
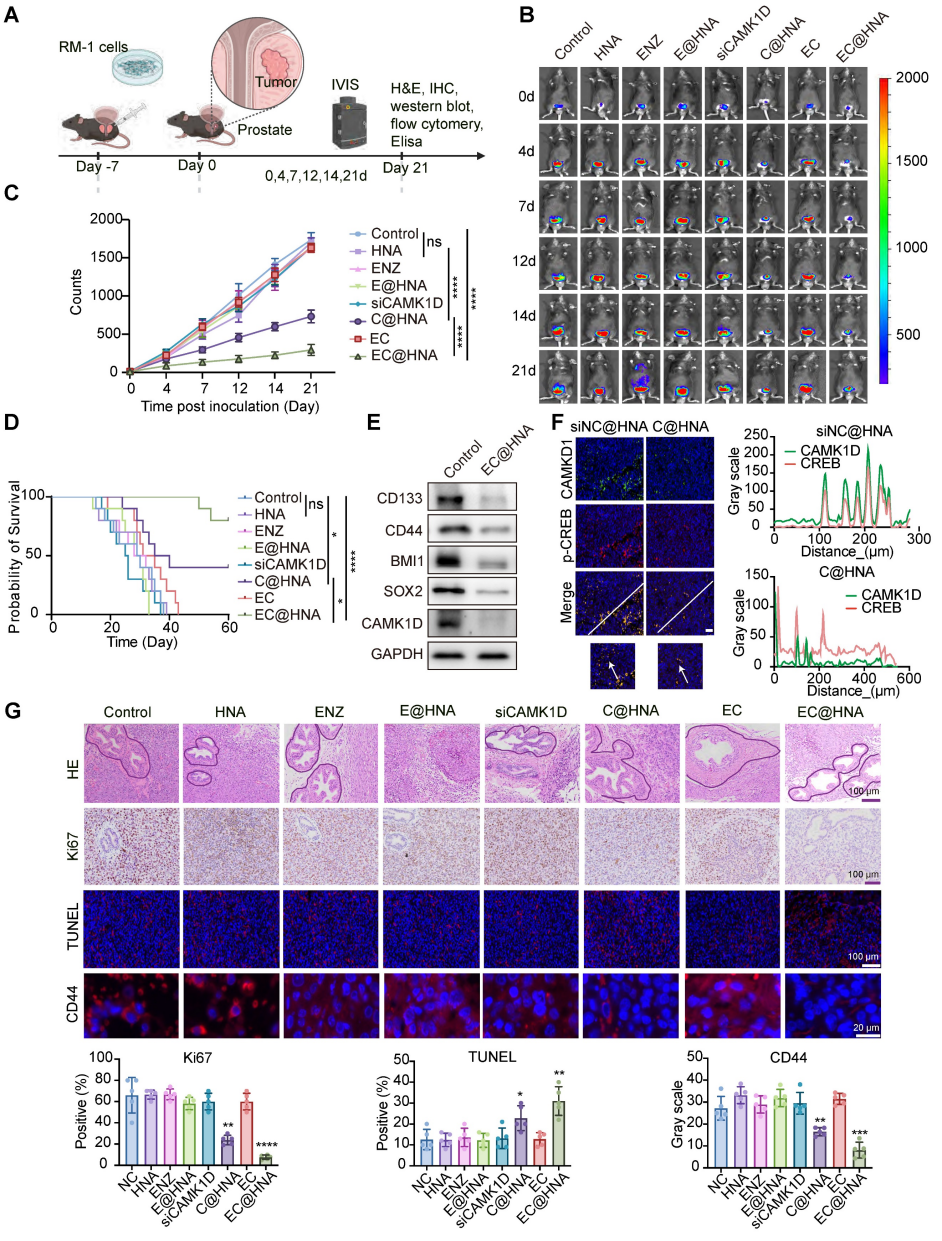
EC@HNA effectively reverses ENZR *in vivo*. (A) Schematic representation of the *in vivo* experimental procedure. Orthotopic prostate tumors were established by injecting 1 × 10⁵ luciferase-labeled RM-1 cells per mouse. After tumor fluorescence was detected, mice were randomly assigned to eight groups and received various formulations of siCAMK1D (3 μg/mouse) or ENZ (5 mg/kg) via tail vein injection every three days. (B) Bioluminescent images showing tumor growth in mice subjected to different treatments using Living Image software. (C) Quantification of bioluminescence signals from tumor-bearing mice after various treatments (n=5). (D) Survival rates of subcutaneous RM-1 tumor-bearing mice treated with the indicated therapies (n = 10). (E) Western blot analysis of stemness markers and CAMK1D expression in PCa xenografts. (F) Immunofluorescence staining of CAMK1D and phosphorylated CREB in mouse tumor tissues. (G) Immunofluorescence images depicting Ki67, CD44, and TUNEL staining in PCa tissue sections. The areas outlined with black borders indicate normal prostatic glandular tissue, whereas the remaining regions correspond to tumor tissue. Data were assessed for normal distribution using the Shapiro-Wilk test, and statistical comparisons were performed using two-sided Student's *t*-test.^ *^P < 0.05, ^**^P < 0.01, ^***^P < 0.001, ^****^P < 0.0001.

To assess the therapeutic efficacy of EC@HNA in treating ENZR-PCa, we established *in situ* tumor models and monitored tumor growth using an IVIS system (Figure 5A-B). The cNP dosage was optimized through *in vitro* dose-response and *in vivo* pilot studies for effective gene silencing with minimal cytotoxicity, ENZ dosing was based on established preclinical studies and pilot experiments. Quantitative analysis of bioluminescence signals revealed that the EC@HNA treatment group exhibited the lowest tumor fluorescence intensity, indicating the most potent inhibitory effect compared with the other seven treatment groups (Figures 5C and S7A). Throughout the experiment, no significant changes in body weight or organ toxicity were observed across all groups, indicating the satisfactory biosafety of EC@HNA (Figure S7B-C). Additionally, the survival rate of mice treated with EC@HNA was markedly higher than that of mice in the other groups (Figure 5D). Histological analysis using Hematoxylin and Eosin (HE) staining and immunofluorescence imaging of *in situ* tumor samples showed a substantial reduction in the number of tumor nodules and degree of nuclear condensation, and an increase in apoptotic cell rates in the EC@HNA-treated group (Figures 5G and S7D). Western blot results revealed a significant decrease in stemness markers in the EC@HNA group, suggesting that EC@HNA directly impaired the stem-like properties of ENZR PCa cells (Figure 5E). Furthermore, TUNEL staining of prostate tumor sections identified a higher apoptosis level in the EC@HNA group (Figure 5F). Collectively, these results indicated that EC@HNA exerted potent tumor suppression in ENZR PCa cells while maintaining a favorable safety profile, highlighting its potential as an effective therapeutic strategy.

### CAMK1D mediates immune evasion in ENZR PCa model

**Figure 6:**
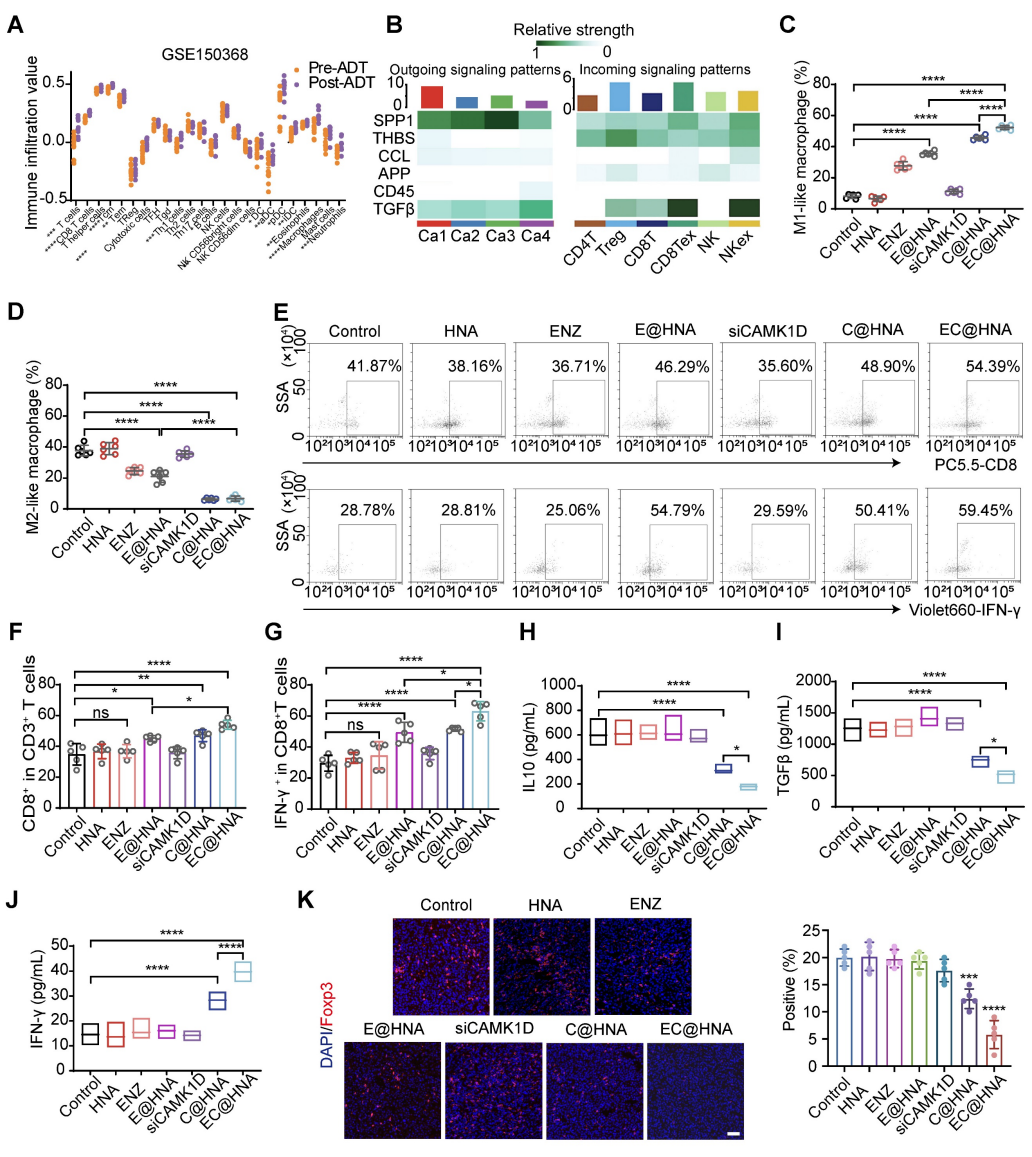
*In vivo* immune activation and exertion of potent anti-ENZR PCa efficacy with EC@HNA. (A) Using the ssGSEA method in R, we scored the correlation between ADT and immune infiltration using the GSE150368 dataset. (B) Effects of CAMK1D on immune infiltration in PCa. (C) Percentage of M1-like phenotype (CD45^+^CD11b^+^F4/80^+^CD86^+^) and (D) M2-like phenotype (CD45^+^CD11b^+^F4/80^+^CD206^+^) after treatment with different formulations (n=5). (E) Flow cytometric analyses of CD8^+^ T cells and IFN-γ^+^ CD8^+^ T cells. (F) Percentage of CD8^+^ T cells in CD3^+^ T cells (n=5). (G) Percentage of IFN-γ^+^ CD8^+^ T cells (n=5). Levels of (H) IL-10, (I) TGF-β, and (J) IFN-γ secreted by ENZR PCa tissues were measured by ELISA (n=5). (K) Immunofluorescence identifying Treg cells in orthotopic PCa tumors from C57BL/6 mice injected with the indicated RM-1 cells. Scale bar, 50 µm. ^*^P < 0.05, ^**^P < 0.01, ^***^P < 0.001, ^****^P < 0.0001.

Previous research has indicated that ENZ treatment transforms PCa into an immunogenic "hot" tumor, however, this conversion still fails to effectively inhibit tumor proliferation (Figure 6A). CSCs often evade immune surveillance through various mechanisms, fostering an immunosuppressive tumor microenvironment characterized by CD8^+^ T cell exhaustion, M2 macrophage polarization, and increased Treg infiltration 7. Our findings confirm that CAMK1D is a crucial regulator of stemness in ENZR PCa cells, raising questions about whether CAMK1D contributes to the stemness-mediated immunosuppressive microenvironment. We first analyzed the effects of CAMK1D on immune cell infiltration in PCa and found that CAMK1D was negatively correlated with CD8^+^ T cells (Figure 6B). Moreover, EC@HNA treatment significantly increased the number of tumor-infiltrating M1-like macrophages and reduced the number of M2-like macrophages (Figures 6C-D and S8). *In vivo*, CAMK1D interference reduced Treg infiltration and elevated CD8⁺ T cell levels from 41.87% to 54.89%, accompanied by a substantial increase in IFN-γ⁺ CD8⁺ T cells from 28.78% to 59.45% in PCa tissues (Figures 6E-G, 6K and S9). Moreover, the EC@HNA group exhibited the most significant reduction in IL-10 and TGF-β levels, which are essential for Treg cell differentiation 27, along with the greatest increase in IFN-γ expression (Figure 6H-J). Our results suggest that ENZR PCa cells with low CAMK1D expression following C@HNA treatment remodel the immunosuppressive microenvironment by reprogramming multiple immune cell populations, which may enhance the efficacy of immune checkpoint inhibitors (ICIs) in these patients 28.

### CAMK1D promotes stemness in ENZR cells by enhancing CREB phosphorylation

**Figure 7:**
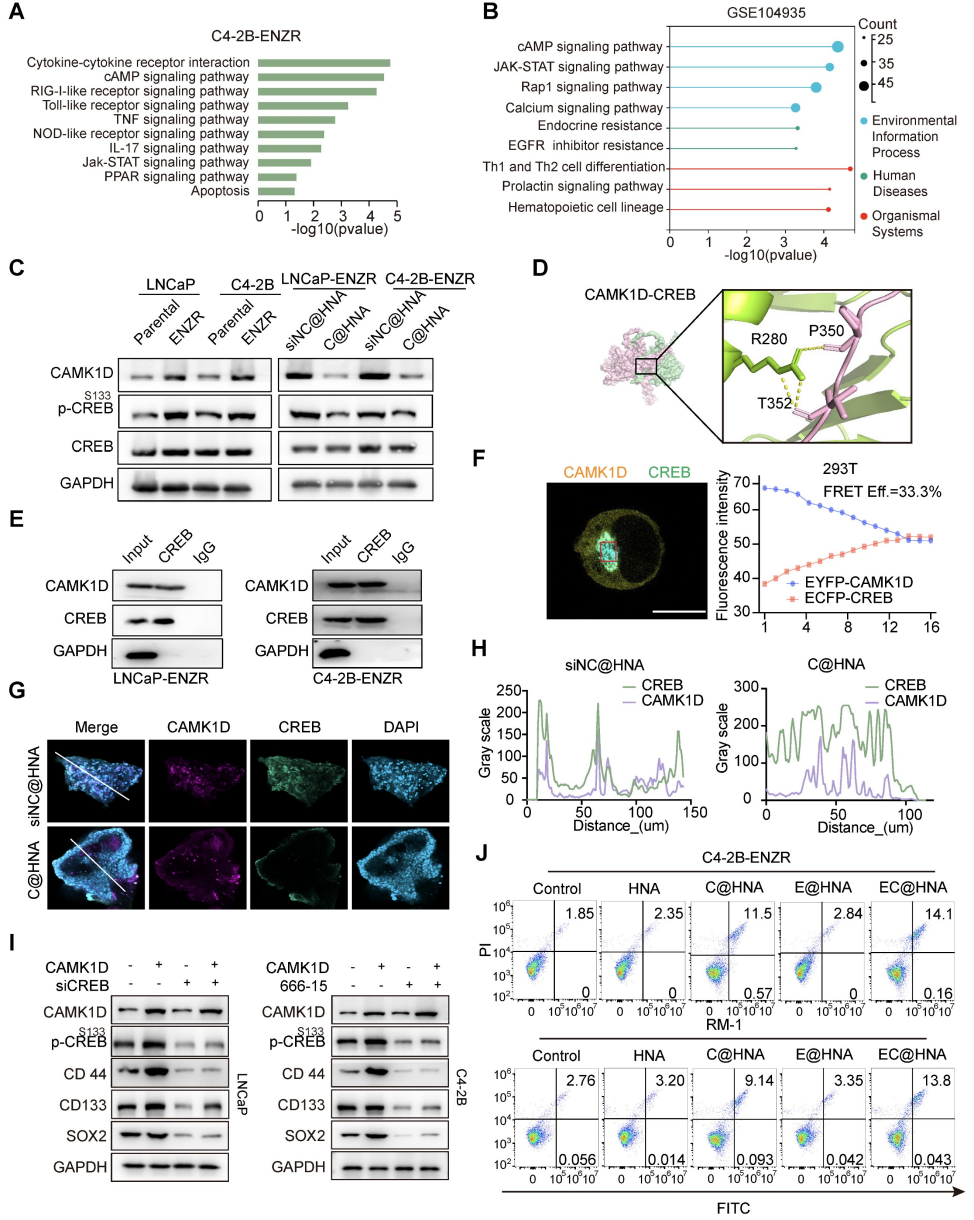
CAMK1D interacts with CREB and modulates its transcriptional activity in ENZR cells. (A) Meta-enrichment analysis summary for 187 significantly co-upregulated genes. (B) Top KEGG pathways enriched (*P* < 0.05) in androgen-deprived therapy-resistant tumors from the GSE104935 dataset. (C) Immunoblot analysis of the indicated proteins in LNCaP-Parental/LNCaP-ENZR and C4-2B-Parental/C4-2B-ENZR cells. The phosphorylation levels of CREB are shown in the indicated cells. LNCaP-ENZR and C4-2B-ENZR cells were transfected with EC@HNA and analyzed by western blot. (D) Molecular dynamics docking analysis predicting potential binding sites and interaction interface between CAMK1D and CREB. (E) Co-IP assays showing the interactions between CAMK1D and CREB. Co-IP assays were performed using LNCaP-ENZR and C4-2B-ENZR cell lysates. (F) Representative FRET measurement of CAMK1D and CREB interaction in 293T cells. FRET was performed on 293T cells co-transfected with FRET sensor pairs: CFP-donor (CREB) and YFP-acceptor (CAMK1D). Fluorescence intensity was detected using a confocal microscope. Scale bar, 10 µm. Fluorescence intensity of CFP and YFP channels before and during acceptor photobleaching. All images were captured with a confocal microscope at 63 × magnification. (G,H) Immunofluorescence staining showing co-localization of CAMK1D (green) and CREB (red) in PCa organoids and xenografts. (I) Western blot analysis of stemness marker expression following CAMK1D overexpression or CREB knockdown in LNCaP and C4-2B cells. (J) Flow cytometry analysis of apoptosis in LNCaP and C4-2B cells subjected to different treatments. Data are presented as mean ± SD from three independent experiments.

Given the critical role of the androgen receptor (AR) signaling pathway in PCa, the effect of CAMK1D on AR and AR-V7 protein levels was examined and found to have no significant impact (Figure S10A). To better understand CAMK1D-induced phenotypic changes, we performed GSEA on the GEO104935 dataset and C4-2B-ENZR siNC/siCAMK1D transcriptome arrays. Interestingly, we identified several high-scoring gene sets (*P* < 0.05, FDR < 0.1) functionally associated with CSCs and immune response-related pathways, such as AMPK signaling pathway and JAK-STAT signaling pathways (Figure 7A-B). Previous studies have demonstrated that ADT induces CREB transcriptional activity, thereby promoting ENZR expression in PCa cells 29. The transcriptional activity of CREB is also modulated by CAMK1D 30. Thus, we hypothesize that CAMK1D promotes stemness-mediated ENZR expression in PCa cells by enhancing CREB transcriptional activity. First, we confirmed that phosphorylation of CREB was significantly elevated in ENZR cells compared to that in parental cells, whereas its phosphorylation levels were significantly reduced in the EC@HNA group compared to those in the HNA group (Figure 7C). To further investigate the relationship between CAMK1D and CREB, we performed a molecular dynamics docking analysis of the structures of the two proteins and identified potential binding sites (Figure 7D). Subsequently, Co-IP and immunofluorescence results revealed interaction and co-localization in the nucleus, which was further confirmed by FRET assays (Figures 7E-F, and S10B). Immunofluorescence analysis of the organoids also demonstrated a consistent reduction in CAMK1D and CREB expression in the C@HNA group compared to the control group (Figure 7G-H). Furthermore, CAMK1D knockdown by EC@HNA significantly decreased the expression of stem cell-associated genes and promoted apoptosis in ENZR cells (Figure 7I-J). Using the JASPAR and PROMO databases, we identified several potential binding sites for CREB in the promoters of BCL2, SOX2, CD44 and NR4A1 (Figure S10C-D). Then, ChIP-PCR assays confirmed the direct binding of CREB to these regions for the transcriptional expression of stemness-related proteins (Figure S10E). These results indicate that CAMK1D promotes a stem-like cell phenotype in ENZR cells, possibly by modulating CREB transcriptional activity.

## Discussion

CSCs, characterized by their self-renewal and differentiation potential, are key contributors to tumor heterogeneity and represent a formidable barrier to effective cancer therapy 31. In ENZR PCa cells, CAMK1D is notably upregulated, likely through the activation of stemness-associated signaling pathways; its expression is negatively correlated with responses to immunotherapy. Therefore, the targeted knockdown of CAMK1D is a promising strategy for overcoming ENZR and enhancing anti-tumor immune responses. However, the clinical applications of siRNAs are limited by their short half-life, poor cellular uptake, rapid enzymatic degradation, and instability, highlighting the need for an advanced delivery system to achieve therapeutic success.

To address these challenges, we developed a novel nanoactivator, EC@HNA, which exhibits high biocompatibility, low immunogenicity, inherent tumor-targeting properties, and enhanced drug-loading capacity. Compared with both the Control and FC@NA groups, FC@HNA treatment significantly increased cellular fluorescence (*P* < 0.05), indicating that HA modification facilitated CD44-mediated uptake. This result is consistent with its established role in promoting targeted nanoparticle internalization 32, 33 and supports the functional advantage of HNA in promoting targeted delivery. At the beginning of the treatment, elevated CD44 expression facilitated robust HA-mediated accumulation of EC@HNA in resistant tumors. As therapy progresses and CD44 levels decrease, nanoparticles continue to achieve intratumoral enrichment through residual CD44 binding and passive targeting via the Enhanced Permeability and Retention (EPR) effect, thereby maintaining superior delivery and therapeutic efficacy compared with free molecules. Pulmonary retention primarily reflects first-pass effects and alveolar macrophage uptake, which are features characteristic to nanoparticle pharmacokinetics regardless of the targeting ligands. Splenic accumulation may reflect both CD44^+^ cell enrichment and nonspecific nanoparticle uptake because a similar distribution was observed for D@NA. Similarly, HNA-based nanoparticles may reach the thymus and bone marrow through a combination of CD44-dependent and passive pathways. Although not examined in this study, we agree that exploring bone tropism in metastatic PCa is a promising direction for future studies. Using this platform, we achieved efficient downregulation of CAMK1D, significantly increasing the sensitivity of ENZR cells to ENZ and improving therapeutic outcomes in ENZR PCa.

Recent studies have highlighted the involvement of CAMK1D in cancer, particularly in the context of drug resistance 11; however, its precise role in mediating resistance mechanisms remains unclear. Among PCa stemness-associated markers, CD44, CD133, and NR4A1 are closely associated with resistance to AR inhibitors including ENZ 34. We demonstrated that CAMK1D enhanced the transcriptional activity of CREB, leading to increased expression of stemness markers. Prior studies have established that stemness markers, particularly CD44 and CD133, are essential for maintaining tumor cell survival and contribute to poor therapeutic outcomes 35-37. Activated CREB binds to the promoters of stemness-related genes, such as CD44 and SOX2, driving their transcriptional upregulation and maintaining a stem cell-like phenotype that confers therapeutic tolerance 5. Consistent with previous findings, CREB was identified as a pivotal transcription factor that mediates adaptive responses to AR blockade. CREB activation promotes AR reactivation under androgen-deprived conditions 38 and induces transcriptional reprogramming toward stem cell-like or neuroendocrine phenotypes 29. Cellular stemness and EMT are key features of lineage plasticity in PCa, which enable tumor cells to adapt to AR pathway inhibition 39. Therefore, the CAMK1D-mediated enhancement of CREB activity observed in our study may contribute to ENZR, primarily by reinforcing lineage plasticity and sustaining stem-like transcriptional programs. This mechanistic connection highlights the potential of simultaneously targeting the CAMK1D and CREB pathways to overcome drug resistance and improve therapeutic efficacy in PCa. This finding deepens our understanding regarding the role of CREB in ENZR and opens new avenues for the development of therapies that target CAMK1D or its downstream signaling networks, thus offering the potential to enhance clinical outcomes for patients.

PCa, traditionally categorized as an immune-cold tumor, presents significant challenges for immunotherapy, and its resistance to ENZ further exacerbates this issue by fostering an immunosuppressive tumor microenvironment 28, 40. Bioinformatic analysis indicated that CAMK1D expression was negatively correlated with CD8^+^ T cell infiltration, suggesting its role in immune evasion. Single-cell analysis revealed an increase in T-cell infiltration in the ENZ-treated group, whereas the numbers of natural killer (NK) cells and M2 macrophages were reduced. Notably, *in vivo* experiments demonstrated that EC@HNA treatment effectively decreased Treg cell levels while increasing CD8^+^ T cell proportions in PCa tissues, accompanied by downregulation of IL-10 and TGF-β expression. Although IL-10 was initially classified as a T helper 2 (TH2)-type cytokine, subsequent evidence has associated it with the Treg cell response 41. TGF-β, a pleiotropic cytokine that bridges pathological immune responses with CSC regulation, plays a critical role in shaping the tumor microenvironment 42. It suppresses NK and cytotoxic T cell activity, promotes tumor-associated neutrophils (TANs), and polarizes macrophages toward an immunosuppressive phenotype, all of which contribute to immune evasion 43. Collectively, these findings indicate that EC@HNA not only mitigates cancer stemness to overcome drug resistance, but also reactivates anti-tumor immunity, providing a dual mechanism for the effective treatment of ENZR PCa. Our results highlight a novel therapeutic strategy with substantial clinical potential and lay the groundwork for treatments that precisely target resistance mechanisms while enhancing immune activation in ENZR PCa.

## Conclusion

In this study, we engineered a hyaluronic acid-modified lipid-based nanoactivator (EC@HNA) for the co-delivery of ENZ and siCAMK1D, which was designed to enhance the sensitivity of ENZR PCa cells to T cell-mediated cytotoxicity while mitigating immune evasion driven by stemness factors. The selected drug combination was optimized at a fixed ratio to achieve sustained co-release in vivo, thereby maximizing synergistic effects in ENZR PCa and minimizing dosage and potential toxicity. Our findings revealed that CAMK1D significantly contributes to ENZR by enhancing stemness through CREB activation. EC@HNA inhibited phosphorylated CREB, which downregulated the stemness markers CD44 and CD133 and prevented resistant tumor cells from surviving therapeutic stress. Additionally, EC@HNA suppressed TGF-β expression, thereby attenuating Treg-mediated immunosuppression and reducing immune evasion. Moreover, EC@HNA increased the proportion of M1-like macrophages and decreased the proportion of M2-like macrophages, thus promoting CD8^+^ T cell infiltration and cytotoxic activity in ENZR PCa. The dual role of EC@HNA in overcoming resistance and immune evasion underscores its potential as a therapeutic strategy, suggesting that EC@HNA can improve treatment outcomes by addressing both resistance and immune evasion in PCa.

## Supplementary Material

Supplementary figures and tables.
