# Supplementary material for: Targeting CAMK1D-engineered nanoactivator suppresses cancer stem cell maintenance and immune evasion in enzalutamide-resistant prostate cancer
Source: Theranostics. 2026 Jan 1;16(6):2918–35. doi: 10.7150/thno.120826 (PMC12775825; doi:10.7150/thno.120826)
Supplement: Supplementary file 1 — Supplementary figures and tables. [file thnov16p2918s1.pdf]

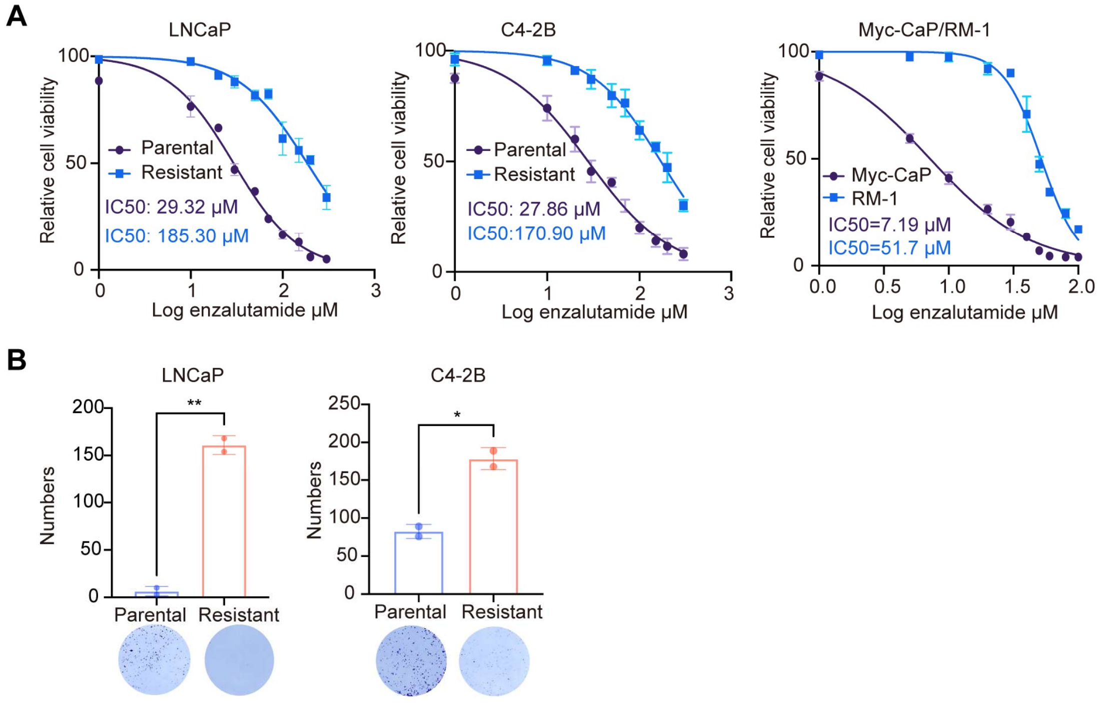

Figure S1. Validation of ENZR cell lines and evaluation of transfection efficiency. (A) LNCaP and C4-2B parental and ENZR cells, along with androgen-sensitive Myc-CaP and androgen-insensitive RM-1 murine prostate cancer cells, were each seeded 2000 cells per well in a 96-well plate and exposed to escalating concentrations of ENZ. Cell viability was quantified after 5 days by measuring absorbance. (B) 600 cells were seeded in six-well plates and treated with 20 μM ENZ. The culture medium and drug were refreshed every 7 days, and after approximately 15 days, cells were fixed and stained for observation. Data are presented as mean ± SD. $^{*}P < 0.05$, $^{**}P < 0.01$.

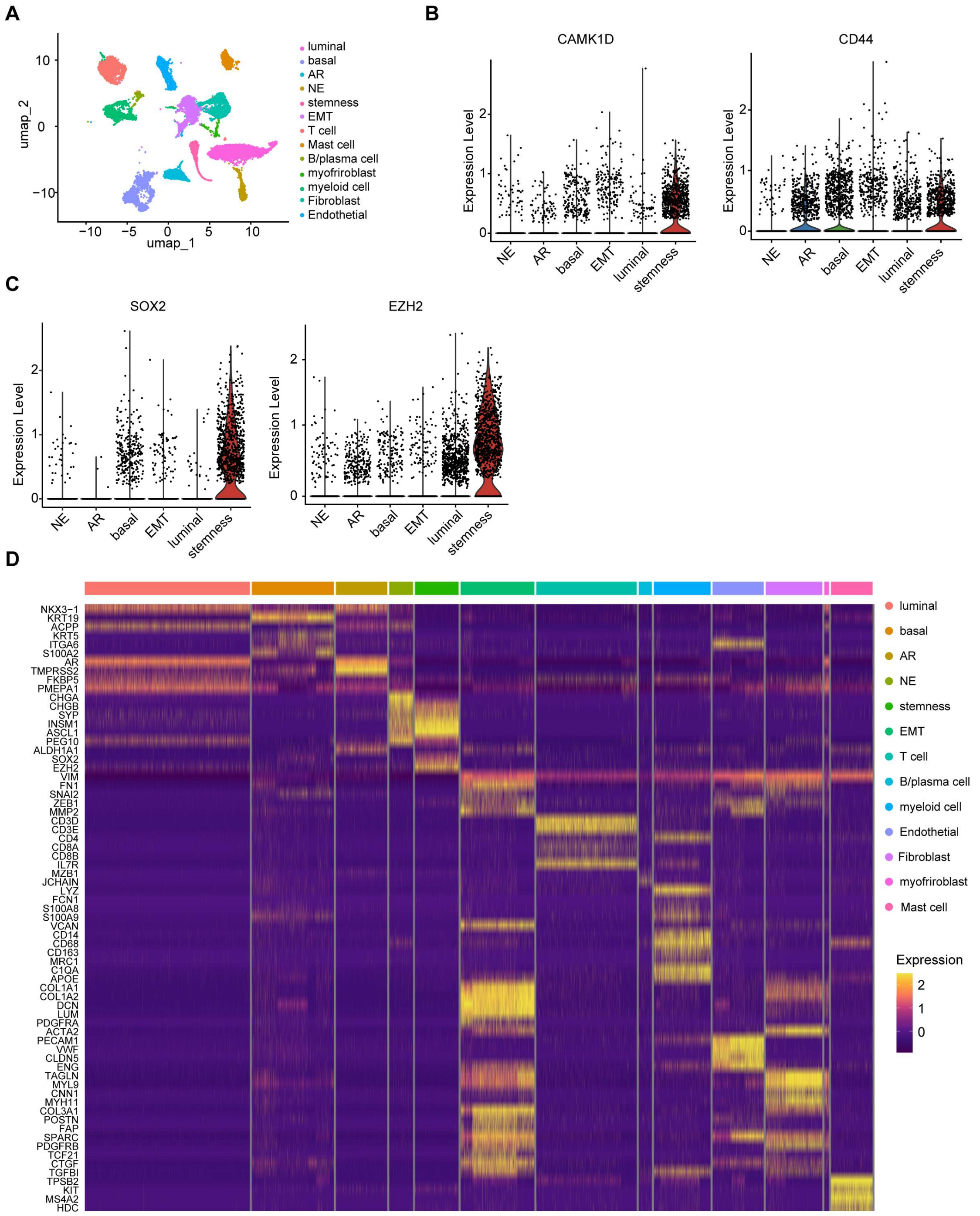

Figure S2. ScRNA-seq of human PCa (GSE137829). (A) Umap visualization of single-cell clusters identified from the GSE137829 dataset. (B-D) Expression landscapes of CAMK1D, CD44, SOX2, and EZH2 across different cell clusters, showing their enrichment within the stemness-associated populations.

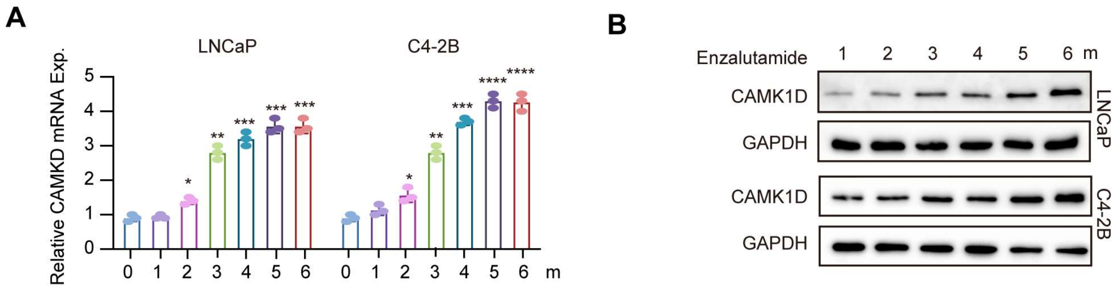

Figure S3. Validation of ENZR cell lines and assessment of CAMK1D expression dynamics. (A, B) In LNCaP and C4-2B cells were treat with 10 μM ENZ, and RNA and protein were collected at the indicated time points. CAMK1D mRNA and protein expression levels were subsequently quantified. $^{*}P < 0.05$, $^{**}P < 0.01$, $^{***}P < 0.001$, $^{****}P < 0.0001$.

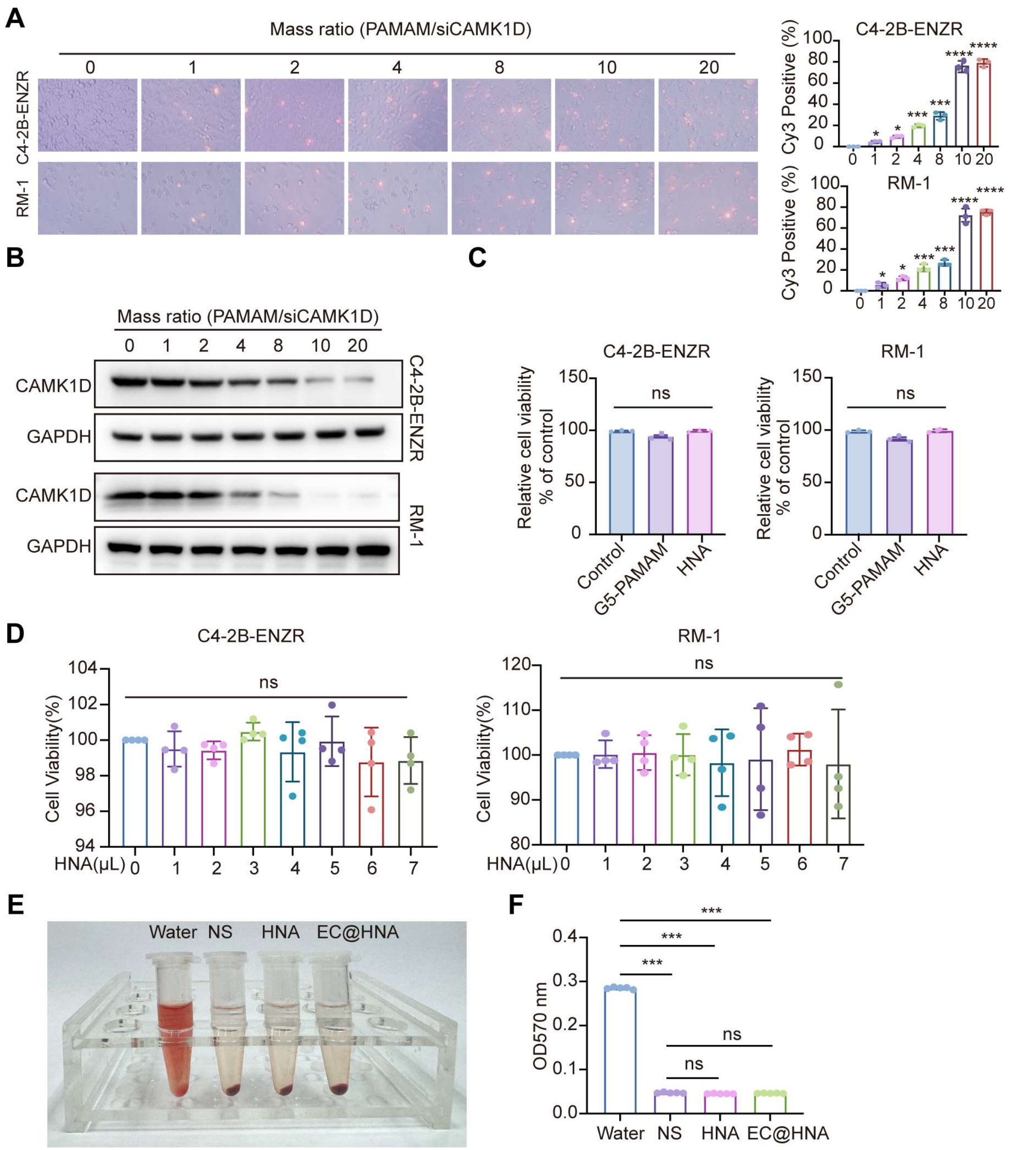

Figure S4. Cytotoxicity and biosafety evaluation of various formulations. (A) Cy3 fluorescence microscopy images of sNP complexes assembled at different mass ratios. (B) Western blotting analysis of CAMK1D expression following transfection with sNP complexes at varying mass ratios. (C) C4-2B-ENZR and RM-1 cells were treated with G5-PAMAM or HNA for 48 h, and cell viability was evaluated using the CCK-8 assay. (D) *In vitro* cytotoxicity of HNA in C4-2B-ENZR and RM-1 cells at the indicated concentrations. (E, F) Hemolytic activity of various formulations (n=5). Data are expressed as mean ± SD. NS represents normal saline; ns, not statistically. $^{*}P < 0.05$, $^{***}P < 0.001$, $^{****}P < 0.0001$.

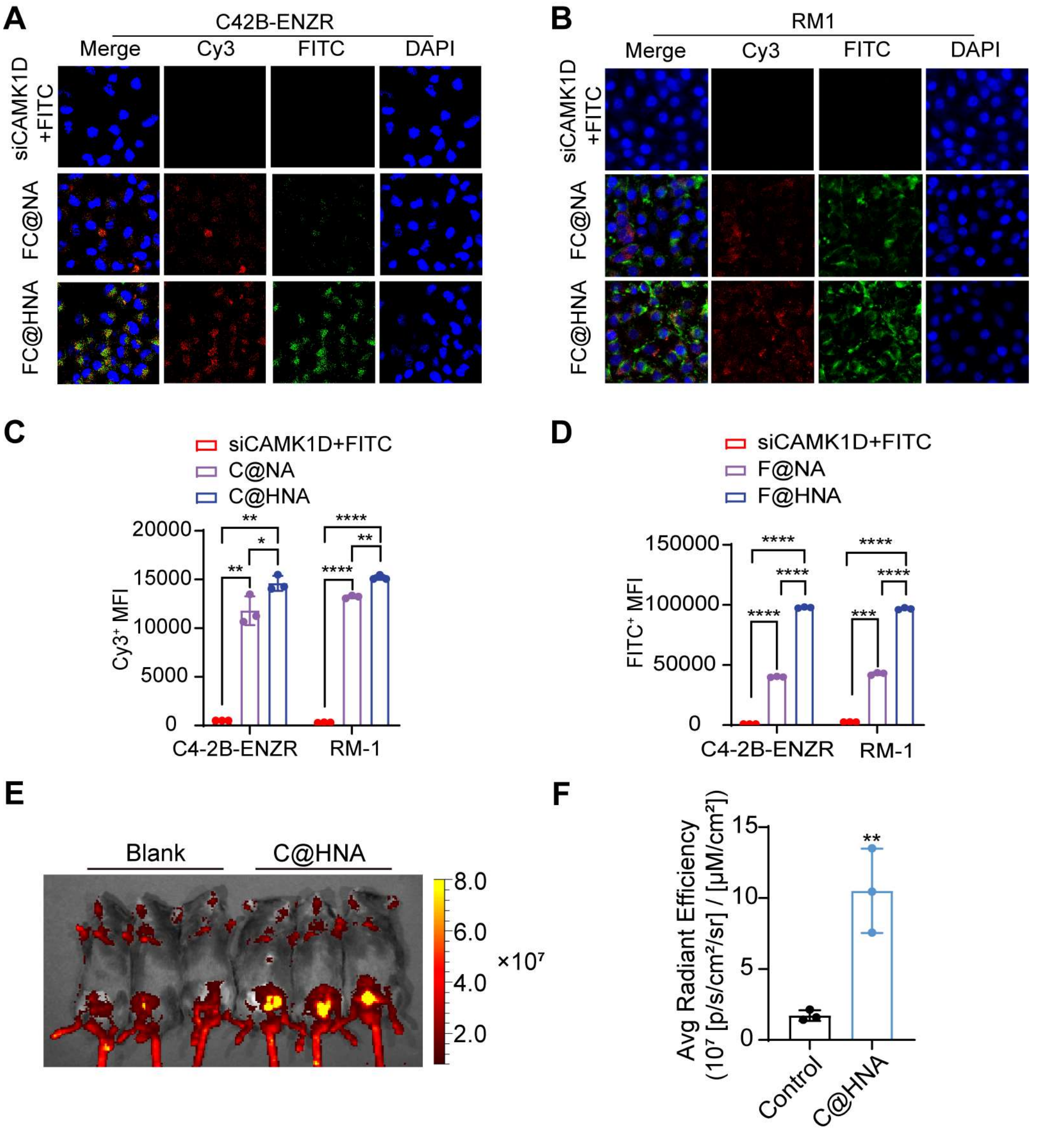

Figure S5. Cellular uptake of HNA in ENZR PCa cells. (A, B) Confocal fluorescence imaging of C4-2B-ENZR and RM-1 cells treated with siCAMK1D+FITC, FC@NA, or FC@HNA. Scale bar: 20 µm. (C, D) MFI ratios of Cy3$^+$ and FITC$^+$ signals in C4-2B-ENZR and RM-1 cells across various treatment groups, analyzed quantitatively via flow cytometry (n=3). (E, F) *In vivo* IVIS fluorescence imaging and quantification of Cy3 signals in Blank and C@HNA-treated mice (n=3). $^{*}P < 0.05$, $^{**}P < 0.01$, $^{***}P < 0.001$, $^{****}P < 0.0001$.

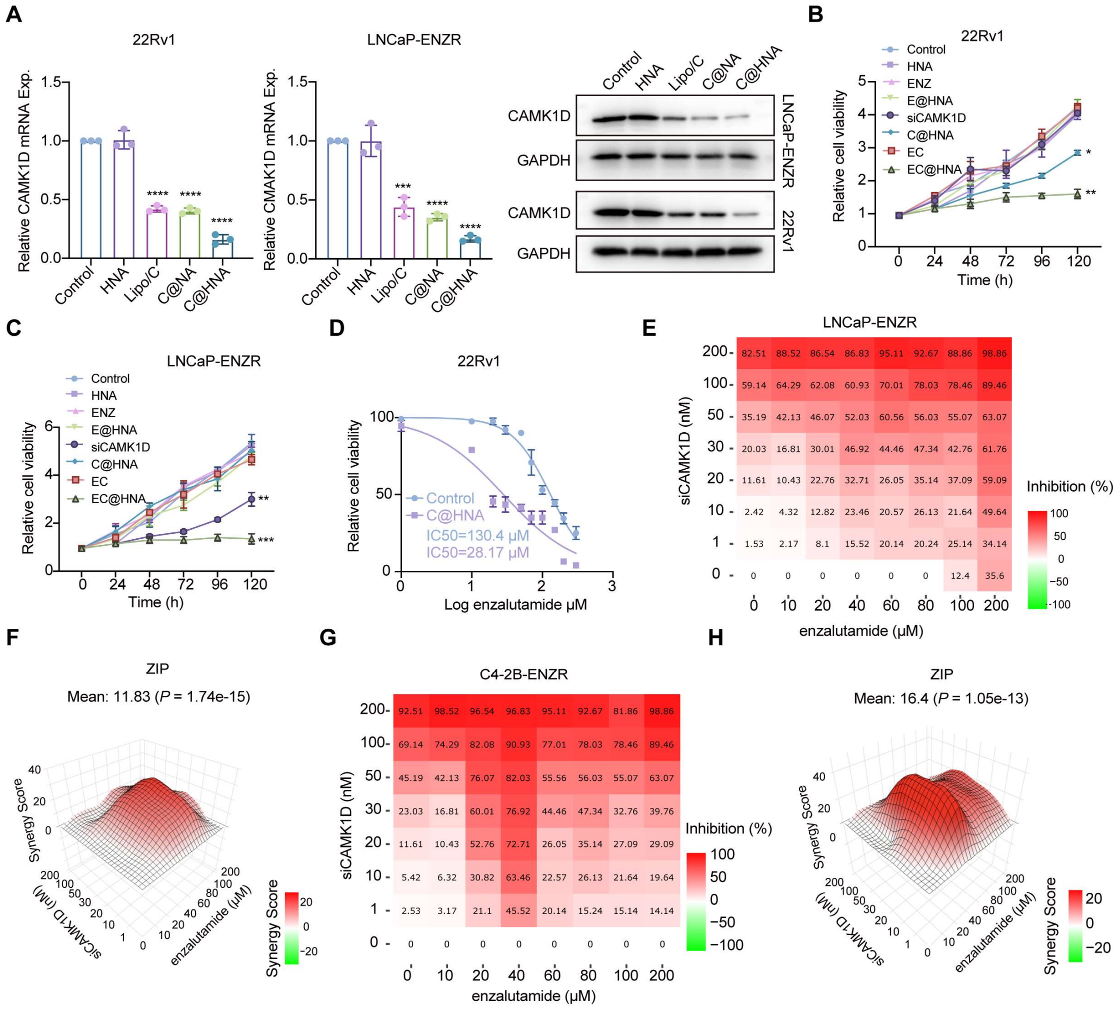

Figure S6. Evaluation of CAMK1D knockdown and synergistic antitumor effects of EC@HNA in ENZR PCa. (A) LNCaP-ENZR and 22Rv1 cells were transfected with HNA, Lipo3000/siCAMK1D (Lipo/C), C@HNA, or EC@HNA. CAMK1D mRNA and protein expression levels were assessed by western blotting. (B, C) Cell proliferation of 22Rv1 and LNCaP-ENZR cells treated with HNA, ENZ, E@HNA, siCAMK1D, C@HNA, EC, or EC@HNA was assessed by CCK-8 assay. (D) 22Rv1 cells pretreated with C@HNA for 24 h were reseeded at 2,000 cells per well and exposed to a gradient of ENZ concentrations for 5 days, after which absorbance was measured to determine viability. Combination index and 3D synergy plots illustrating the synergistic effects of siCAMK1D and/or ENZ at various concentrations on cell proliferation in (E, F) LNCaP-ENZR and (G, H) C4-2B-ENZR cells, analyzed using the SynergyFinder platform (https://synergyfinder.org/). $^{*}P < 0.05$, $^{**}P < 0.01$, $^{***}P < 0.001$, $^{****}P < 0.0001$.

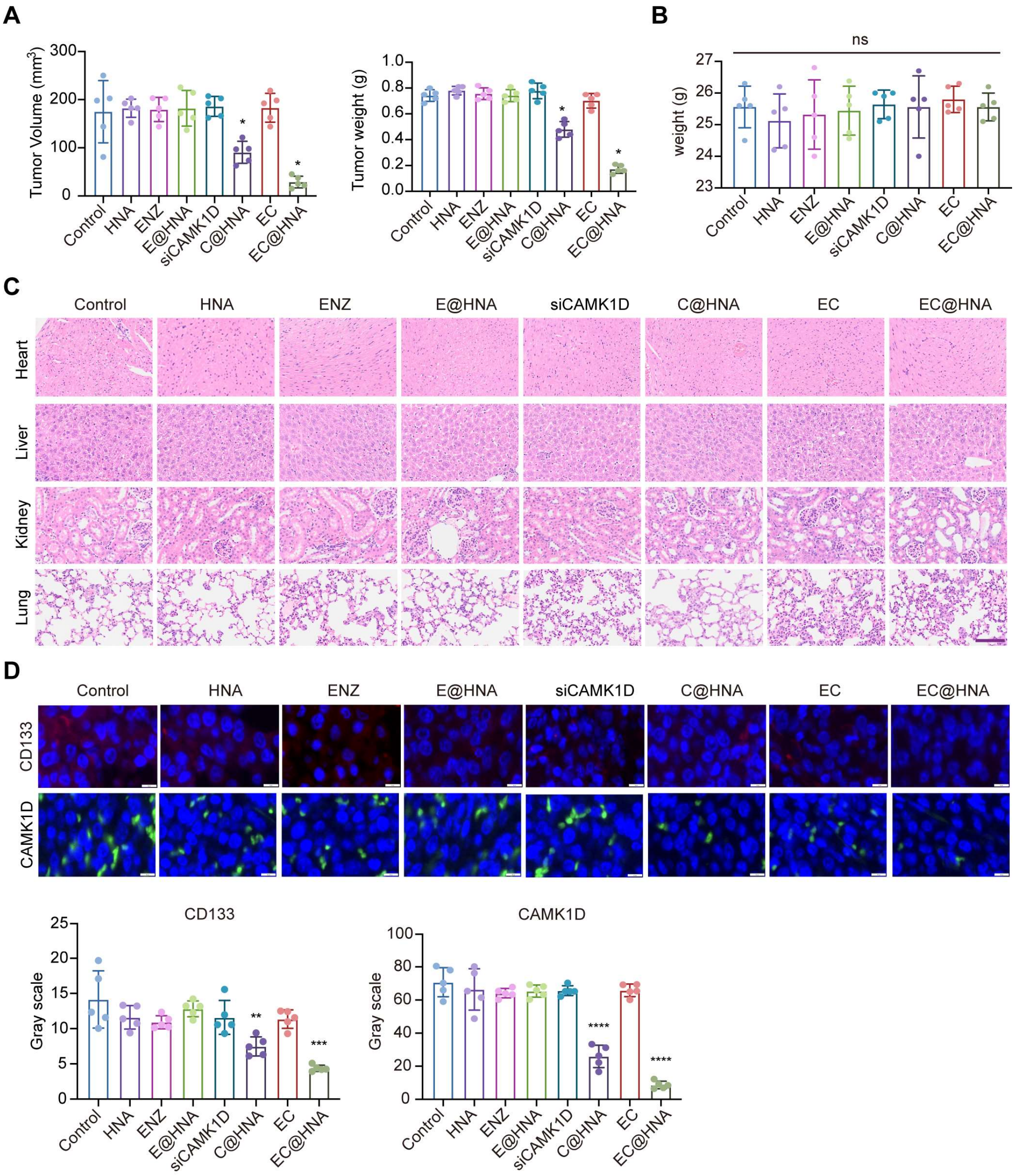

Figure S7. *In vivo* evaluation of tumor growth and biosafety. (A) Tumor size at the experimental endpoint was determined using the product-of-diameter method, and tumor weights were recorded for analysis. (B) Statistical diagram of mouse body weight. (C) Representative H&E-stained sections of major organs (lung, liver, heart, and kidney) following necropsy, confirming the *in vivo* biosafety of HNA. Scale bars, 100 μm. (D) Immunofluorescence staining of primary tumor sections for CD133 and CAMK1D, with quantitative analysis of fluorescence intensities shown below. Scale bars for C, 10 μm. $^{*}P < 0.05$, $^{**}P < 0.01$, $^{***}P < 0.001$, $^{****}P < 0.0001$.

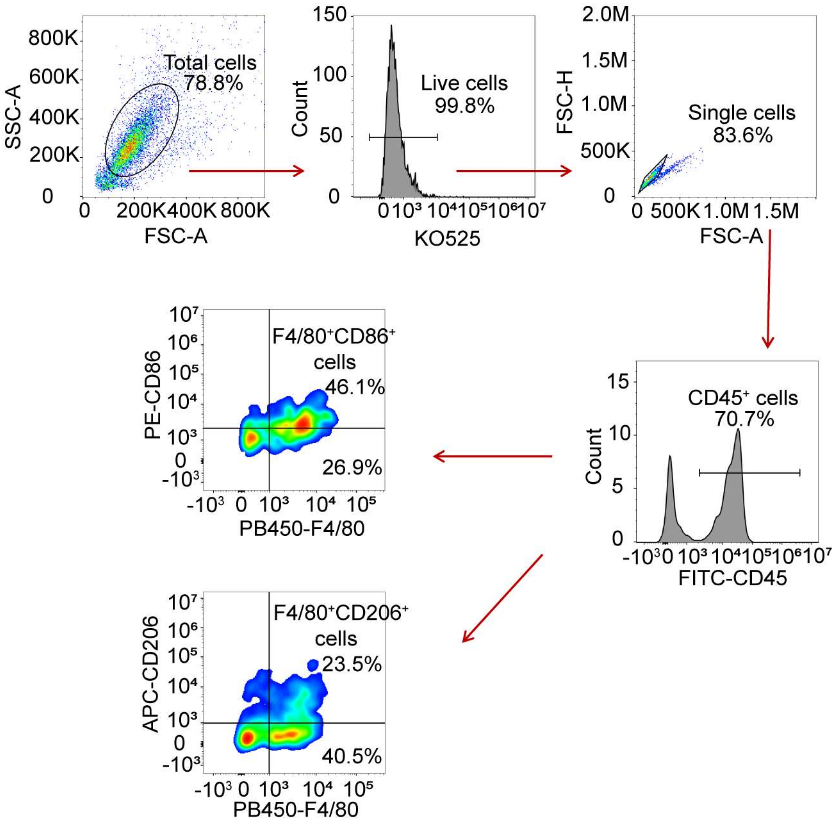

Figure S8. Gating strategies for identifying M1-like and M2-like macrophage subsets.

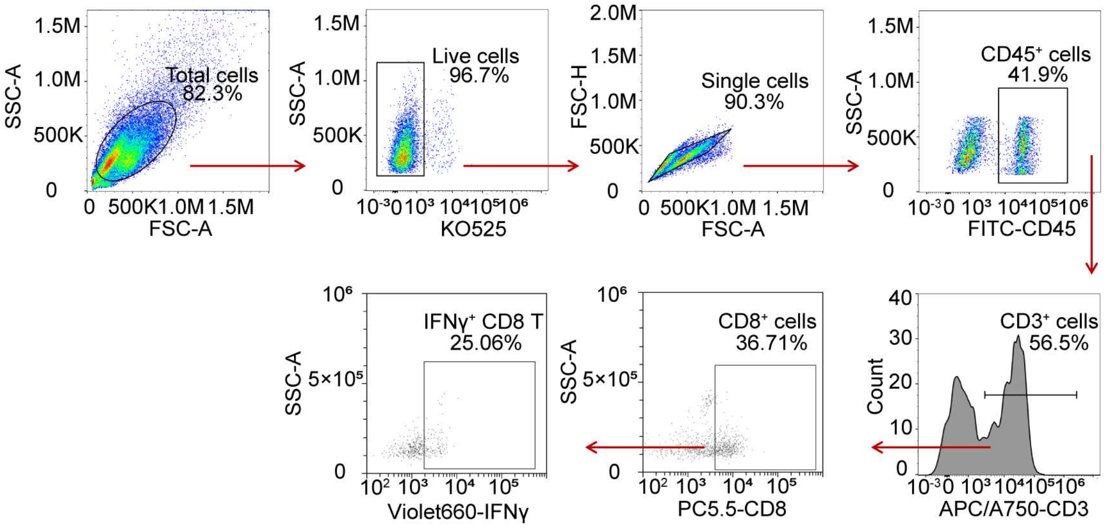

Figure S9. Gating strategies for $CD8^+$ T cells and IFN-γ^+ $CD8^+$ T cells.

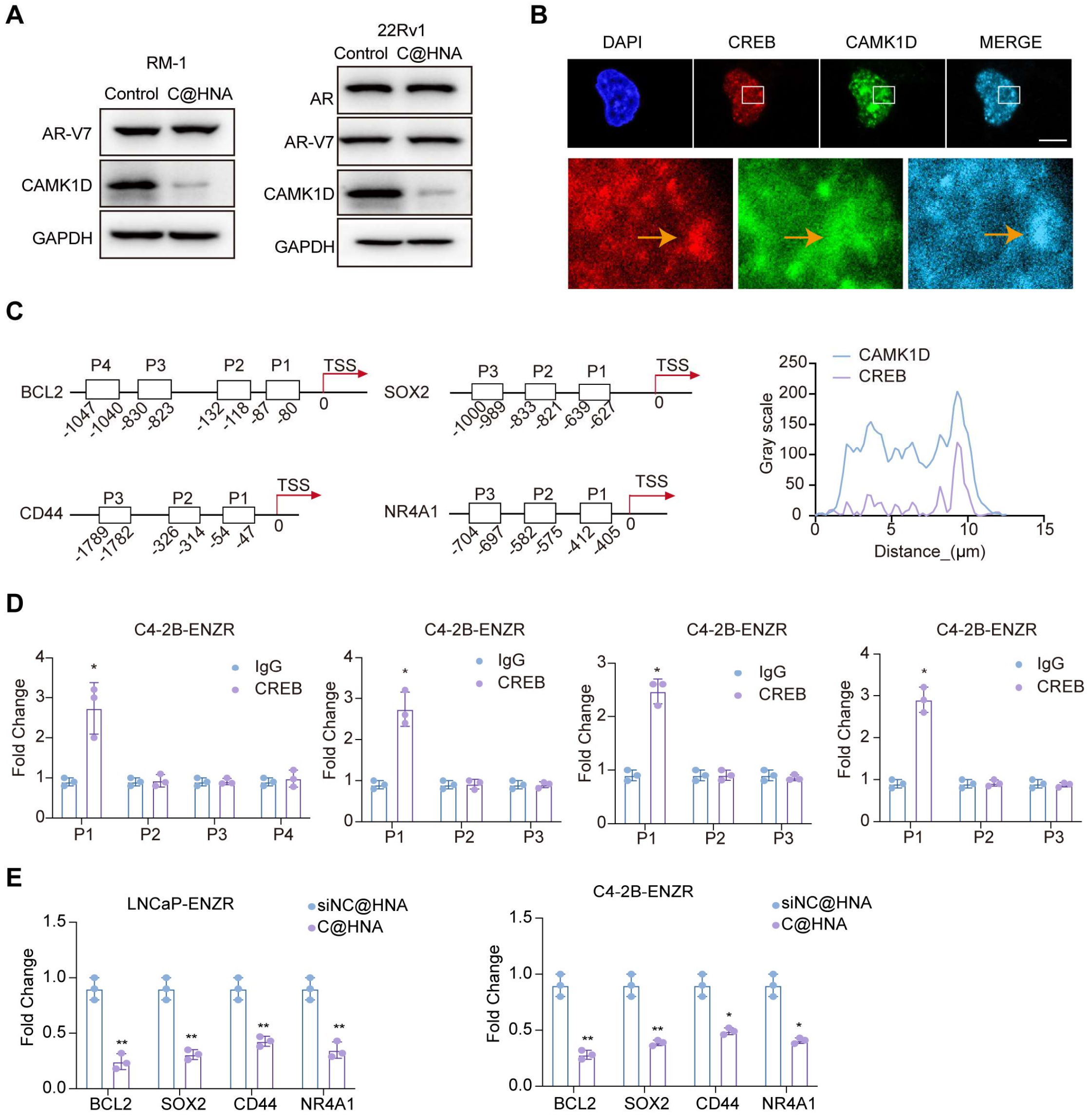

Figure S10. CAMK1D modulates CREB phosphorylation and transcriptional activity. (A) 22Rv1 and RM-1 cells were treated with C@HNA for 48 h, followed by total protein extraction and western blot analysis to assess CAMK1D, AR, and AR-V7 expression levels. (B) Immunofluorescence assays showing nuclear co-localization of CAMK1D and CREB. The bar graph below quantifies the fluorescence co-localization intensity. (C) Design a schematic of CREB transcriptional regulation primers and ChIP PCR result statistics. (D) ChIP-PCR analysis of CREB recruitment onto the BCL2, SOX2, CD44 and NR4A1 promoter in LNCaP-ENZR and C4-2B-ENZR cells. Purified rabbit IgG was used as a negative control. (E) RT-PCR analysis demonstrating downregulation of stemness-associated genes upon EC@HNA treatment. $^{*}P < 0.05$, $^{**}P < 0.01$.

Supplementary Table 1. Antibodies used in this study.

Antibody	Cat no.
GAPDH	ab181602; Abcam
CAMK1D (WB)	ab172618; Abcam
CAMK1D (IF)	ab198165; Abcam
CD44	15675-1-AP; Proteintech
CD133	18470-1-AP; Proteintech
SOX2	ab97959; Abcam
CREB	12208-1-AP; Proteintech
CREB	ab178322; Abcam
p-CREB	9198S; Cell Signaling Technology
BMI1	6964T; Cell Signaling Technology
β-catenin	8480S; Cell Signaling Technology
P63	ab124762; Abcam
Ki67	
Flag	14793S; Cell Signaling Technology
IgG	2729S; Cell Signaling Technology

Supplementary Table 2. Sequence of primers used in this study.

Gene	Forward	Reverse
**RT-qPCR**		
CAMK1D	GTCCACAGAGACCTCAAGCC	GTTTCTGGGCGAGGACTTCA
BCL-2	GGTGAACTGGGGGAGGATTGT	AGAGACAGCCAGGAGAAATCAAAC
SOX2	GCTACAGCATGATGCAGGACCA	TCTGCGAGCTGGTCATGGAGTT
OCT4	CCTGAAGCAGAAGAGGATCACC	AAAGCGGCAGATGGTCGTTTGG
CD44	CCAGAAGGAACAGTGGTTTGGC	ACTGTCCTCTGGGCTTGGTGTT
CD133	CACTACCAAGGACAAGGCGTTC	CAACGCCTCTTTGGTCTCCTTG
Nanog	CTCCAACATCCTGAACCTCAGC	CGTCACACCATTGCTATTCTTCG
NR4A1	GGACAACGCTTCATGCCAGCAT	CCTTGTTAGCCAGGCAGATGTAC
NR4A3	ACTGCCCAGTAGACAAGAGACG	GTTTGGAAGGCAGACGACCTCT
GAPDH	GTCTCCTCTGACTTCAACAGCG	ACCACCCTGTTGCTGTAGCCAA
**ChIP-qPCR**		
BCL2	GGGCAACATAGCAAAAGCCATCT	CCATACTGGCCAGGCTGG
SOX2	TGAGTAAGGGTAGACCAGGGG	TTGTTGTCGCTACACGGAGT
CD44	TCCTGAATCCATGCTGTTCGT	AGCAGGCCATTACCATGCAA
NR4A1	AGACAGAAAGGAAGCTGAGG	CGACCTGAGAGTACGCGTG

These primers were purchased from Generalbiol (Anhui, China).
